# Extracellular ATP drives tryptophan metabolism and aryl hydrocarbon receptor activation to promote cellular senescence

**DOI:** 10.1016/j.jbc.2026.113094

**Published:** 2026-04-27

**Authors:** Daniela Volonte, Steven J. Mullett, Stacy L. Gelhaus, Ferruccio Galbiati

**Affiliations:** 1 Department of Pharmacology & Chemical Biology, University of Pittsburgh School of Medicine, Pittsburgh, Pennsylvania, USA; 2 Health Sciences Mass Spectrometry Core, University of Pittsburgh School of Medicine, Pittsburgh, Pennsylvania, USA

**Keywords:** senescence, ATP, purinergic signaling, tryptophan, AhR

## Abstract

We have shown that extracellular ATP promotes senescence through the activation of the P2Y11 receptor (P2Y11R). The underlying molecular mechanisms remain to be fully established. Synthesis of tryptophan (Trp)-derived indole metabolites is mediated mostly by the gut microbiota. Trp metabolites can activate the aryl hydrocarbon receptor (AhR). Whether eukaryotic cells can generate Trp-derived indoles and their functional significance remains to be fully established. Here, we investigated the role of Trp metabolites and AhR activation in purinergic-mediated senescence of human fibroblasts. We find that ATP activated AhR in a P2Y11R-dependent manner and that AhR activation was necessary for ATP-induced senescence. Stimulation with an AhR agonist was sufficient to induce senescence. Interestingly, depletion of Trp in the conditioned medium inhibited ATP-induced senescence. We show that ATP stimulation upregulated the expression of the L-amino acid oxidase interleukin-4-induced-1, which has been shown to metabolize Trp into indole-3-pyruvate (I3P), in a P2Y11R-dependent fashion. We find that I3P-derived Trp metabolites are upregulated in ATP-induced senescent human fibroblasts and that stimulation with I3P and I3P-derived Trp metabolites was sufficient to promote senescence in these cells. In addition, I3P stimulation activated AhR, and AhR inhibition impaired I3P-induced senescence. Downregulation of interleukin-4-induced-1 inhibited ATP-induced AhR activation and senescence. Finally, we show that conditioned medium derived from senescent lung fibroblasts, which were induced to senesce by I3P treatment, promoted the proliferation of breast cancer cells and their tumorigenic potential. Our study identifies the existence of a novel purinergic dependent signaling pathway that functionally couples Trp metabolism to the development of a premature senescent phenotype in human fibroblasts.

The development of a stable form of cell cycle arrest (1, 2, 3, 4) and the acquisition of a senescence-associated secretory phenotype (SASP), consisting of cytokines, chemokines, and growth factors, are key features of senescent cells (5, 6, 7, 8, 9, 10, 11, 12, 13). Senescent cells are also characterized by an enlarged and flat morphology, and well-defined molecular changes, including increased β-galactosidase activity at pH 6.0, p16 and p21^Waf1/Cip1^ protein expression, p53 activation, and a chronic DNA damage response (14, 15, 16, 17, 18, 19, 20). External stimuli can accelerate the development of a senescent phenotype, a condition known as stress-induced premature senescence (21, 22, 23, 24, 25, 26, 27). It is well-established that senescent cells contribute to aging and the development of age-associated diseases, including cancer, through the acquisition of a SASP (28). Importantly, although only a small number of senescent cells may reside within a given tissue, they compromise tissue integrity and function through their SASP (29). Elimination of senescent cells, either genetically (30, 31) or through the use of senolytics (32, 33, 34), delays age-linked phenotypes and cancer-associated death *in vivo*. The signaling pathways and molecular mechanisms that regulate the acquisition of a senescent phenotype are therefore of upmost importance in both aging and cancer biology.

ATP provides energy to drive a plethora of cellular processes. ATP is also a critical extracellular signal transduction molecule in multiple tissues and cell types (35, 36, 37, 38). Intracellular ATP is released into the extracellular space in response to a variety of stimuli, such as oxidative stress, shear stress, ionizing radiation, stretch, and hypoxia (36, 39, 40). Extracellular ATP activates P2 purinergic receptors. P2 receptors have been subclassified as either metabotropic P2Y receptors or ionotropic P2X receptors (41). Multiple subtypes exist of both the P2Y and P2X receptors, which are differentially expressed in many organs and tissues (42, 43). P2Y receptors are G-protein coupled receptors that couple mostly to G_q/11_, but also G_i/o_ and G_s_, leading to changes in intracellular levels of Ca^++^ and cAMP (44, 45, 46). P2X receptors are ligand-gated ion channels that allow the flux of mostly Ca^++^, Na^+^, and K^+^ ions (47, 48, 49).

We have recently shown that ATP-dependent activation of P2Y11R promotes premature senescence in human lung fibroblasts (50). We found that oxidative stress induced release of ATP and caused senescence in human lung fibroblasts. Inhibition of P2 receptors limited oxidative stress-induced senescence, while stimulation with exogenous ATP promoted premature senescence. Pharmacological inhibition of P2Y11R inhibited premature senescence induced by either oxidative stress or ATP, while stimulation with a P2Y11R agonist was sufficient to induce cellular senescence. The prosenescent signaling pathways downstream of P2Y11R activation remain to be fully identified.

Tryptophan (Trp) metabolism plays a crucial role in multiple physiological and pathological processes, such as immune regulation, neurotransmission, and tumor progression (51, 52). Trp is an essential amino acid. As such, it cannot be synthesized by the human body and must be obtained through the diet. Trp is primarily metabolized *via* the kynurenine pathway, which leads to the production of several bioactive metabolites, including kynurenine (Kyn), kynurenic acid (KynA), and quinolinic acid (53). Indoleamine 2,3-dioxygenase 1 (IDO1) catalyzes the initial step in the kynurenine pathway (54). Alternative metabolic routes of Trp degradation involve the synthesis of serotonin and melatonin, which control mood regulation and circadian rhythms (55). Interestingly, the gut microbiota convert Trp into indoles, which can activate the aryl hydrocarbon receptor (AhR) and regulate mucosal immunity and epithelial integrity (56, 57, 58). Moreover, Trp-derived indoles can be produced by glioblastoma cells (59). Whether purinergic signaling promotes cellular senescence by regulating Trp metabolism remains virtually unexplored.

AhR is a ligand-activated transcription factor that belongs to the basic helix-loop-helix-PER-ARNT-SIM subgroup of the basic helix-loop-helix superfamily of transcription factors (60). The classical role of AhR is to regulate responses to environmental toxins (61). Upon activation, AhR translocates to the nucleus where, by modulating the expression of genes involved in xenobiotic metabolism, immune function, and cell proliferation, it regulates both detoxification and disease pathogenesis (62).

In the present study, we find that stimulation of P2Y11R by ATP promotes senescence through Trp metabolite-dependent AhR activation. Collectively, these findings offer new insights into the molecular mechanisms that functionally connect purinergic signaling, Trp metabolism, and the AhR pathway to the development of a premature senescent phenotype, a cellular process with significant implications for aging, age-related diseases, and cancer. Our study also provides evidence that Trp-derived indoles can be produced by and have a functional role in eukaryotic cells.

## Results

### Extracellular ATP activates AhR in a P2Y11R-dependent manner

We have recently found that P2Y11R activation by ATP promotes senescence in human lung fibroblasts (50). Data show that AhR activity can be modulated by calcium-dependent signaling (63, 64), which is downstream of P2YR. AhR activation has been linked to senescence (65, 66, 67). Thus, to begin to investigate the prosenescent molecular mechanism(s) downstream of purinergic signaling, we asked if AhR was a target of ATP-mediated P2Y11R activation. First, we show that treatment of WI-38 human diploid lung fibroblasts with ATP concentrations ranging from 10 μM to 1.5 mM for 10 days induced senescence (Fig. S1*A*) in these cells without causing any cell death (Fig. S1*B*). Since 1.5 mM ATP induced the highest degree of senescence, we chose to use 1.5 mM ATP stimulation in all subsequent experiments. We would like to highlight that high-micromolar to possibly low-millimolar concentrations of extracellular ATP have been found in the tumor microenvironment (TME) (68). Moreover, necrotic cells, which exist within growing tumors, can potentially release millimolar cytosolic ATP in their immediate microenvironment. Thus, 1.5 mM ATP stimulation is representative of elevated local ATP concentrations that are pathologically relevant such as within the TME.

**Figure 1:**
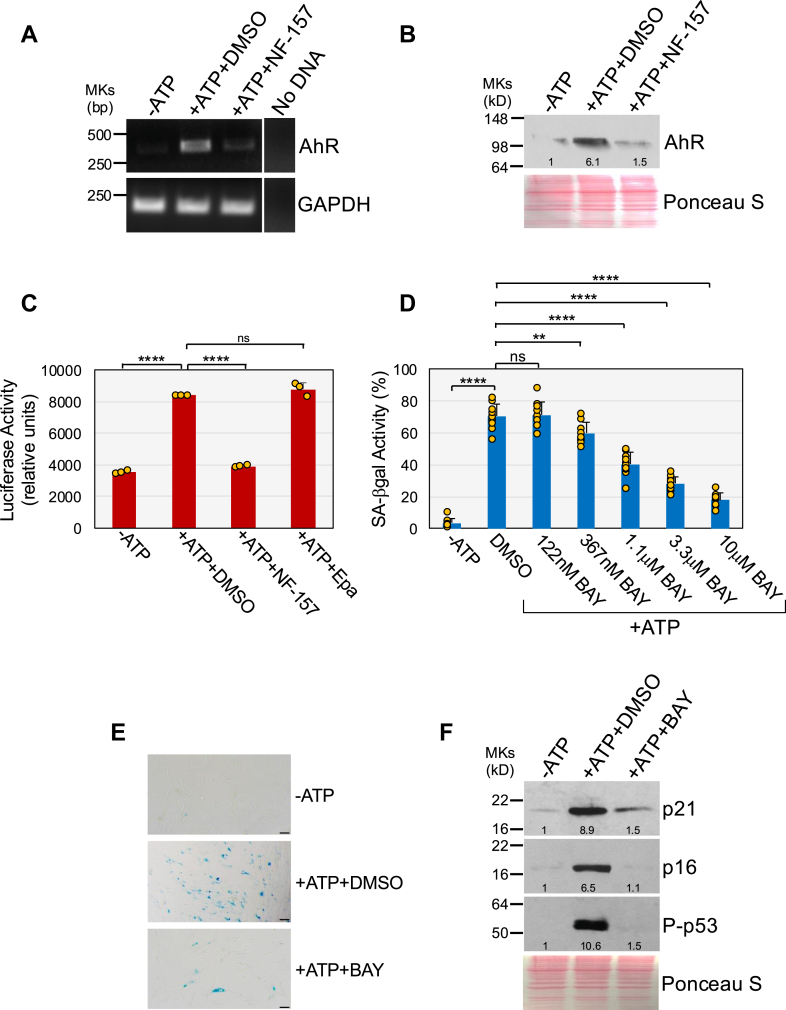
**ATP-induced and P2Y11R-mediated AhR activation promotes premature senescence.***A* and *B*, human diploid WI-38 fibroblasts were treated with 1.5 mM ATP for 1 day in the presence or absence of 40 μM NF-157. Untreated cells were used as control. Cells were collected and AhR mRNA (*A*) and protein (*B*) levels were determined by RT-PCR and immunoblotting analysis, respectively. Expression of GAPDH in (*A*) served as control. Ponceau S staining in (*B*) shows equal total protein loading. Quantification of protein band intensity is shown at the bottom of the blot. *C*, WI-38 fibroblasts were transfected with the XRE-pNL1.3 [SecNLuc] plasmid. After 24 h, cells were treated for 1 day with 1.5 mM ATP in the presence or absence of either NF-157 (40 μM) or Epacadostat (Epa; 15 μM). Untreated cells served as control. Cells were collected and AhR activity was measured by luciferase assay. Values were normalized based on total protein content. *D*, *E*, and *F*, WI-38 cells were treated with 1.5 mM ATP for 10 days in the presence or absence of different concentrations of BAY-218 (BAY). Untreated cells were used as control. (*D*–*E*) Cells were subjected to senescence-associated β-galactosidase activity staining. Quantification is shown in (*D*), representative images are shown in (*E*). In (*F*), cells were collected and p21, p16, and phospho (Ser15)-p53 protein expression was quantified by immunoblotting analysis using specific antibody probes. Ponceau S staining shows equal total protein loading. Quantification of protein band intensity is shown at the bottom of each blot. In *(E*, *F*), 10 μM BAY-218 was used in the experiments. Values in *C* and *D* represent means ± SD; statistical comparisons were made using 1-way ANOVA with *post hoc* test. ∗∗<0.01, ∗∗∗∗<0.0001. The scale bar represents 50 μm. AhR, aryl hydrocarbon receptor; P2Y11R, P2Y11 receptor; XRE, xenobiotic response element.

Then, we treated WI-38 fibroblasts with 1.5 mM ATP in the presence or absence of NF-157, a selective P2Y11R antagonist. Cells were collected and the expression level of AhR was determined by RT-PCR and immunoblotting analysis. We show that ATP upregulated both AhR mRNA (Fig. 1*A*) and AhR protein expression (Fig. 1*B*) in WI-38 fibroblasts. Inhibition of P2Y11R dramatically impaired ATP-induced upregulation of both AhR mRNA and AhR protein expression (Fig. 1, *A* and *B*). We also find that ATP stimulated a luciferase-based construct carrying a xenobiotic response element in a P2Y11R-dependent manner (Fig. 1*C*). We have previously shown that P2Y11R/phospholipase C (PLC)-mediated release of calcium from intracellular stores mediates ATP-induced senescence (50). Consistent with these results, either intracellular calcium chelation with BAPTA-AM or PLC inhibition with U73122 impaired AhR activation by ATP (Fig. S1*C*). Importantly, cell viability was not affected by ATP stimulation in the presence or absence of either NF-157 (Fig. S2*A*), BAPTA-AM, or U73122 (Fig. S2*B*). Thus, AhR is upregulated and activated in human lung fibroblasts through P2Y11R signaling following ATP stimulation.

### ATP promotes senescence through AhR activation. AhR activation is sufficient to induce premature senescence

**Figure 2:**
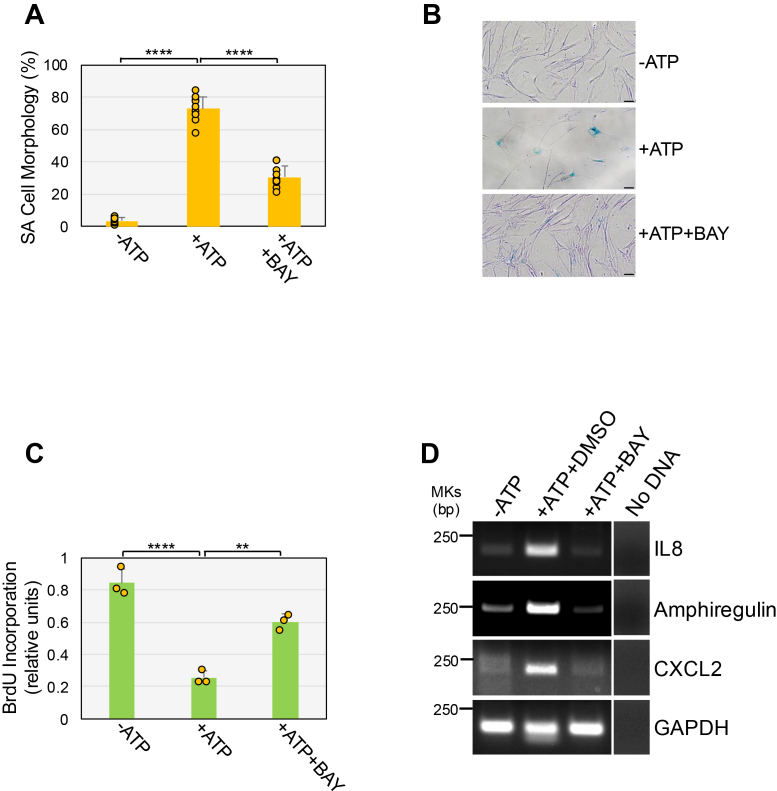
**ATP promotes senescence in an AhR-dependent manner.** WI-38 cells were treated with 1.5 mM ATP for 10 days in the presence or absence of BAY-218 (10 μM). Untreated cells were used as control. *A* and *B*, SA cell morphology was quantified in (*A*), representative images are shown in (*B*). *C*, cell proliferation was quantified by BrdU incorporation assay. *D*, cells were collected and RNA extracted. IL-8, Amphiregulin, and CXCL2 mRNA levels were determined by RT-PCR analysis. Expression of GAPDH served as control. Values in A and C represent means ± SD; statistical comparisons were made using 1-way ANOVA with *post hoc* test. ∗∗<0.01, ∗∗∗∗<0.0001. The scale bar represents 50 μm. AhR, aryl hydrocarbon receptor; SA, senescence-associated.

**Figure 3:**
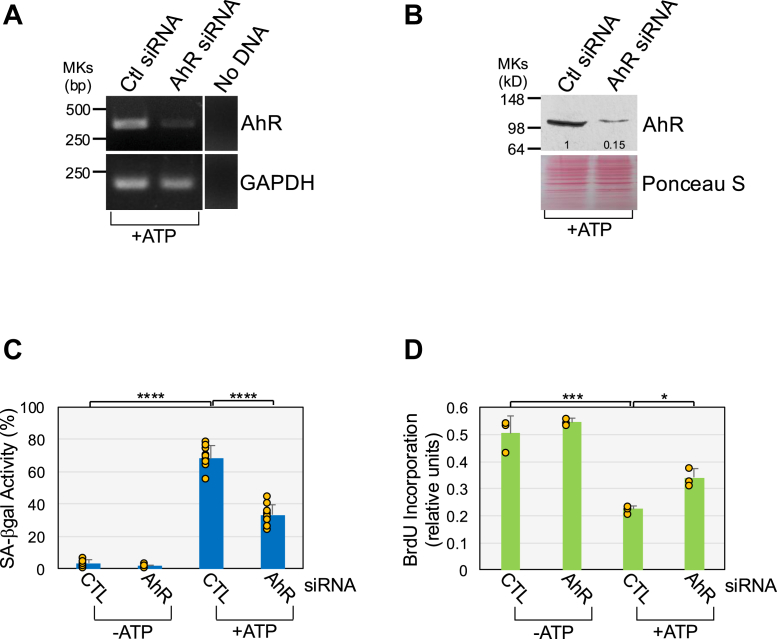
**Downregulation of AhR inhibits ATP-induced senescence.** WI-38 fibroblasts were transfected with either CTL or AhR siRNA. After 24 h, cells were treated with 1.5 mM ATP for either 1 (*A*, *B*) or 10 (*C*, *D*) days. Untreated cells served as control. In (*A*), AhR mRNA expression was quantified by RT-PCR analysis using AhR-specific primers. GAPDH expression served as control. In (*B*), AhR protein level was determined by immunoblotting analysis. Ponceau S staining shows equal total protein loading. Quantification of protein band intensity is shown at the bottom of the blot. In (*C*), cellular senescence was quantified by senescence-associated β-galactosidase activity staining. In (*D*), cell proliferation was quantified by BrdU incorporation assay. Values in C and D represent means ± SD; statistical comparisons were made using 2-way ANOVA with *post hoc* test. ∗<0.05, ∗∗∗<0.001, ∗∗∗∗<0.0001. AhR, aryl hydrocarbon receptor; CTL, control.

We asked if ATP-mediated AhR activation promotes cellular senescence. We first show that treatment of WI-38 fibroblasts with BAY-218, a potent and selective AhR inhibitor, inhibited ATP-induced AhR activation (Fig. S2*C*). Then, WI-38 fibroblasts were treated with ATP for 10 days in the presence or absence of BAY-218. We find that ATP-mediated upregulation of the senescence markers senescence-associated β-galactosidase (SA-β-gal) activity (Fig. 1, *D* and *E*), p21, p16, phospho-p53 (Fig. 1*F*), and senescence-associated cell morphology (flat and large cell morphology; Fig. 2, *A* and *B*) was suppressed in ATP-treated cells in which AhR was inhibited. Consistent with these results, ATP-induced inhibition of cell proliferation was impaired in BAY-218-treated cells (Fig. 2*C*). Senescent cells acquire a senescent-associated secretory phenotype. Consistent with the results above, we find that ATP stimulation upregulated the expression of the SASP factors IL-8, amphiregulin, and CXCL2 (Fig. 2*D*). AhR inhibition with BAY-218 impaired ATP-induced upregulation of IL-8, amphiregulin, and CXCL2 (Fig. 2*D*). As a control, we show that cotreatment of ATP with BAY-218 did not affect cell viability (Fig. S2*D*). In support of these findings, downregulation of AhR mRNA expression by siRNA (Fig. 3*A*) downregulated AhR protein levels (Fig. 3*B*) and suppressed ATP-mediated upregulation of SA-β-gal activity (Fig. 3*C*) and inhibition of cell proliferation (Fig. 3*D*). We confirmed these data using a different AhR-specific siRNA (AhR siRNA #2) in Fig. S3, *A*–*D*, where we show that downregulation of AhR mRNA (Fig. S3*A*) and protein expression (Fig. S3*B*) inhibited ATP-induced upregulation of SA-β-gal activity (Fig. S3*C*) and p21 expression (Fig. S3*D*). We have shown in a previous study that both oxidative stress and UV-C light promote the release of ATP (50). Consistent with the findings above, AhR inhibition also impaired the development of both oxidative stress- and UV-C light-induced senescence in WI-38 fibroblasts (Fig. S3*E*).

**Figure 4:**
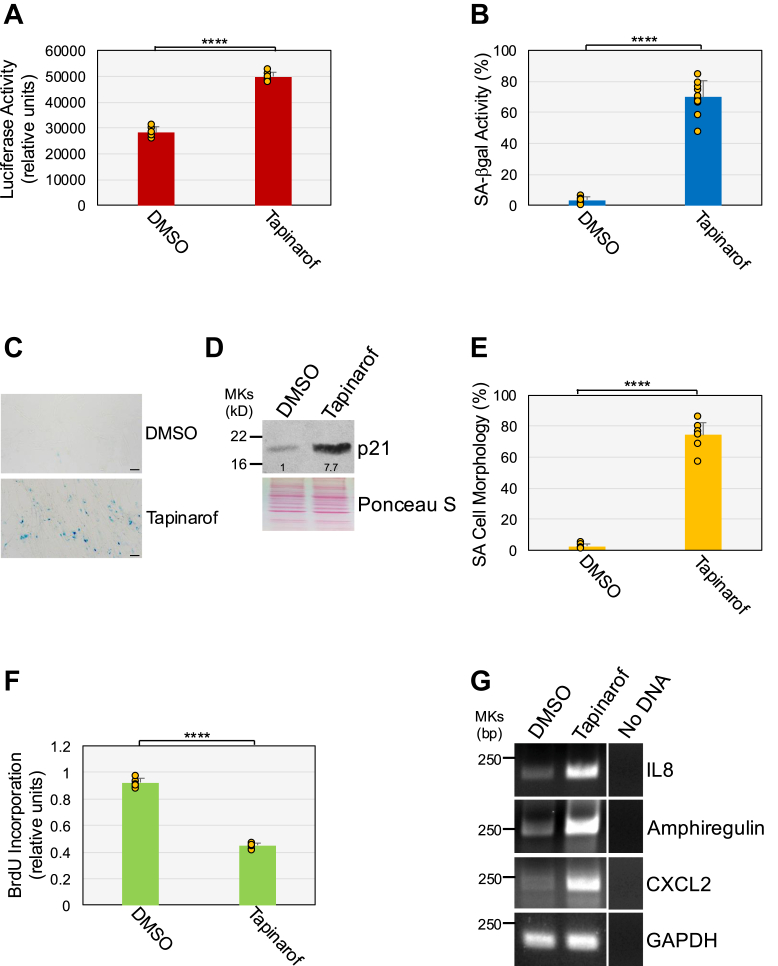
**Activation of AhR is sufficient to promote senescence.***A*, WI-38 fibroblasts were transfected with the XRE-pNL1.3 [SecNLuc] plasmid. After 24, cells were treated for 1 day with tapinarof (9 μM). Untreated cells served as control. Cells were collected and AhR activity was measured by luciferase assay. Values were normalized based on total protein content. *B*, *C*, *D*, *E*, *F*, and *G*, WI-38 fibroblasts were treated with tapinarof (9 μM) for 10 days. Untreated cells were used as control. *B*, and *C*, cells were stained to detect senescence-associated β-galactosidase activity. Quantification is shown in (*B*), representative images are shown in (*C*). *D*, cells were collected, and cell lysates were subjected to immunoblot analysis using an antibody probe specific for the senescence marker p21. Ponceau S staining shows equal total protein loading. Quantification of protein band intensity is shown at the bottom of the blot. *E*, SA cell morphology was quantified. *F*, cell proliferation was quantified by BrdU incorporation assay. *G*, the expression levels of the senescence markers IL-8, amphiregulin, and CXCL2 were determined by RT-PCR analysis. GAPDH expression served as control. Values in *A*, *B*, *E*, and *F* represent means ± SD; statistical comparisons were made using the student’s *t* test. ∗∗∗∗<0.0001. The scale bar represents 50 μm. Of note, we have used the dose of 9 μM tapinarof in our experiments, because it was based on our preliminary dose-response experiments, the lowest dose that provided the maximal senescent-inducing effect in WI-38 fibroblasts without causing toxicity (not shown). AhR, aryl hydrocarbon receptor; XRE, xenobiotic response element.

Interestingly, treatment of WI-38 fibroblasts with the AhR agonist, tapinarof, was sufficient to activate AhR (Fig. 4*A*), upregulate SA-β-gal activity (Fig. 4, *B* and *C*), p21 protein expression (Fig. 4*D*), and the percentage of cells with a SA cell morphology (Fig. 4*E*), and to inhibit cell proliferation (Fig. 4*F*) in the absence of ATP stimulation. Consistent with these data, tapinarof treatment upregulated the expression of IL-8, amphiregulin, and CXCL2 (Fig. 4*G*). We conclude that activation of AhR mediates ATP-induced senescence and that AhR stimulation *per se* can induce senescence in human lung fibroblasts.

### Trp depletion inhibits ATP-induced senescence

**Figure 5:**
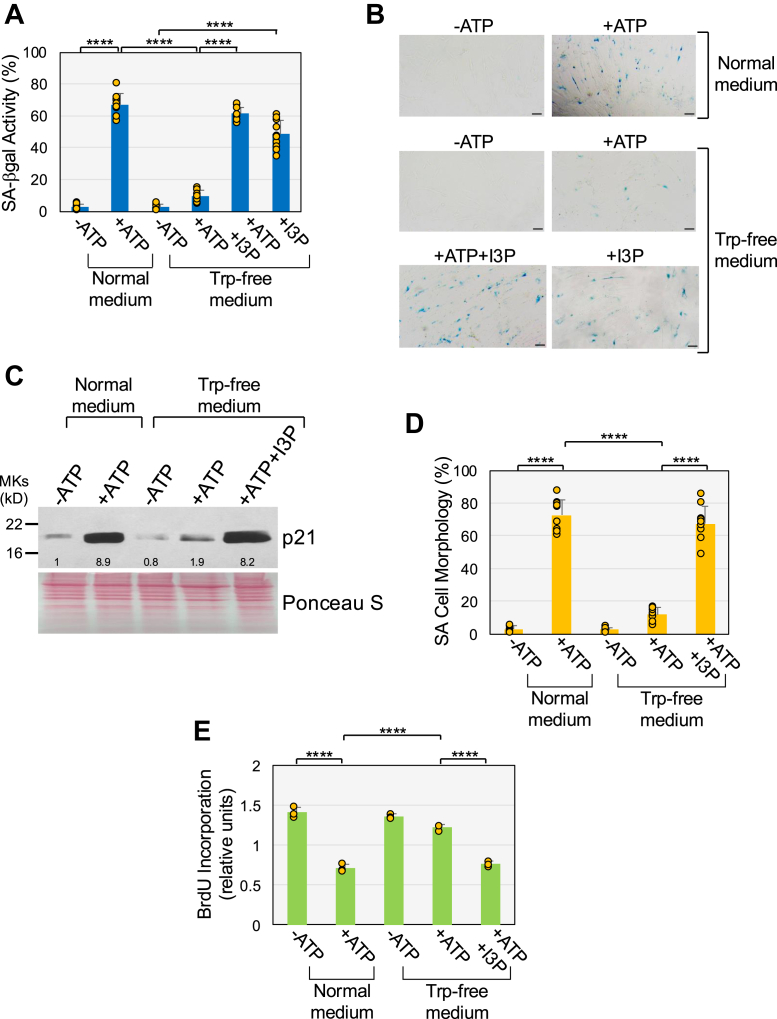
**Tryptophan is necessary for ATP-induced senescence.** Human diploid WI-38 fibroblasts were treated with 1.5 mM ATP for 10 days in normal medium or tryptophan-free (Trp-free) medium. ATP-stimulated WI-38 cells that were cultured in tryptophan-free medium were also treated with 27 μM I3P. *A* and *B*, cells were subjected to senescence-associated β-galactosidase activity staining. Quantification is shown in (*A*), representative images are shown in (*B*). *C*, cells were collected and the expression level of the senescence marker p21 was detected by immunoblotting analysis using p21-specific IgGs. Ponceau S staining shows equal total protein loading. Quantification of protein band intensity is shown at the bottom of the blot. *D*, SA cell morphology was quantified. *E*, cell proliferation was quantified by BrdU incorporation assay. Values in *A*, *D*, and E represent means ± SD; statistical comparisons were made using 2-way ANOVA with *post hoc* test. ∗∗∗∗<0.0001. The scale bar represents 50 μm. SA, senescence-associated.

Since Trp metabolites can activate AhR, we tested if Trp metabolism played a role in ATP-induced senescence. To this end, we determined if a lack of Trp in the culturing medium impaired ATP-induce senescence. We treated with ATP for 10 days WI-38 fibroblasts that were cultured in Trp-free medium. As a control, WI-38 cells were treated with ATP and cultured in normal Trp-containing medium. Cells were collected and cellular senescence was quantified by SA-β-gal activity (Fig. 5, *A* and *B*), p21 protein expression (Fig. 5*C*), SA cell morphology (Fig. 5*D*), and BrdU incorporation assay (Fig. 5*E*). We find that ATP-induced upregulation of SA-β-gal activity, p21 protein expression, SA cell morphology, and inhibition of cell proliferation were suppressed when the cells were cultured in the absence of Trp in the culturing media (Fig. 5, *A*–*E*). Thus, the presence of Trp in the culturing media promotes ATP-induced premature senescence in human diploid fibroblasts.

### Interleukin-4-induced-1 (IL4I1)-dependent Trp metabolites are upregulated in senescent fibroblasts following ATP stimulation

How is Trp metabolized in human lung fibroblasts to mediate AhR activation and senescence following ATP stimulation? Conversion of Trp to Kyn by IDO1 is the main route through which Trp is metabolized in human cells. Thus, we asked if IDO1-mediated conversion of Trp to Kyn promoted ATP-induced senescence. WI-38 human fibroblasts were treated with ATP for 10 days in the presence or absence of Epacadostat, a selective IDO1 inhibitor. We find that IDO1 inhibition did not affect either cell viability (Fig. S2*A*) or ATP-induced senescence, as shown by SA-β-gal activity (Fig. S4*A*) and BrdU incorporation (Fig. S4*B*). In addition, we show that IDO1 inhibition did not impair ATP-induced AhR activation (Fig. 1*C*). As a control for the inhibition of IDO1 by Epacadostat, we show in Fig. S4*C* that treatment of WI-38 cells with Epacadostat significantly inhibited AhR activation induced by Trp stimulation. Finally, we find that treatment with Kyn and its metabolite KynA did not induce senescence in WI-38 cells (Fig. S4, *D* and *E*). As a control for AhR activation by Kyn, we show that KynA stimulation activated AhR in WI-38 lung fibroblasts (Fig. S4*F*). We conclude that IDO1-dependent conversion of Trp to Kyn does not mediate ATP-induced senescence.

**Figure 6:**
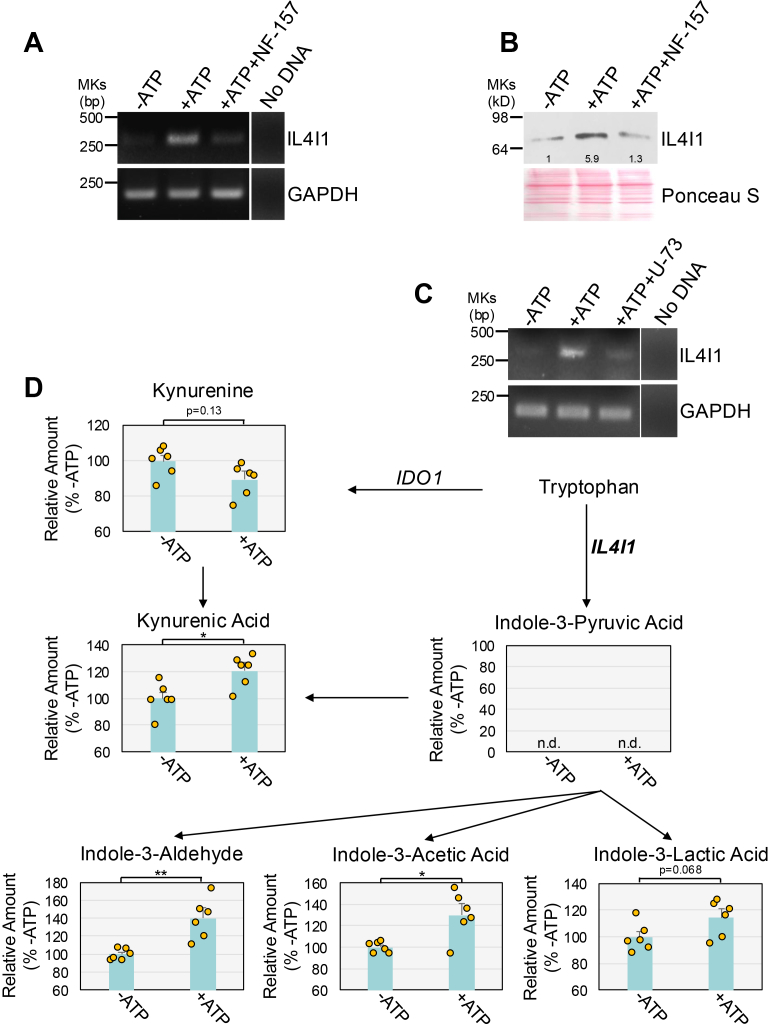
**ATP stimulation promotes the accumulation of IL4I1-dependent and I3P-derived Trp metabolites.***A* and *B*, WI-38 fibroblasts were treated with 1.5 mM ATP for 1 day in the presence or absence of 40 μM NF-157. Untreated cells were used as control. Cells were collected. IL4I1 mRNA (*A*) and protein (*B*) levels were determined by RT-PCR and immunoblotting analysis, respectively. Expression of GAPDH in (*A*) served as control. Ponceau S staining in (*B*) shows equal total protein loading. Quantification of protein band intensity is shown at the bottom of the blot. The Ponceau S staining shown here is the same as in Figure 1*B*, as both panels derive from the same membrane and sample set that was sequentially probed with different antibodies. *C*, WI-38 fibroblasts were treated with 1.5 mM ATP for 1 day in the presence or absence of U-73122 (U73; 3 μM). Untreated cells were used as control. Cells were collected and RNA extracted. IL4I1 mRNA level was determined by RT-PCR analysis using IL4I1-specific primers. Expression of GAPDH served as control. *D*, WI-38 fibroblasts were treated with 1.5 mM ATP for 1 day. Untreated cells were used as control. Cell supernatants were collected and Trp metabolites were identified and quantified by LC-HRMS. The analyte peak area was normalized to the internal standard and then to the protein concentration. Values in D represent means ± SD; statistical comparisons were made using the student’s *t* test. ∗<0.05, ∗∗<0.01. IL4I1, interleukin-4-induced-1; LC-HRMS, liquid chromatography–high-resolution mass spectrometry.

IL4I1L is a secreted L-amino acid oxidase. IL4I1 is mainly expressed withing the human immune system, where it regulates immune cell differentiation and activation (69). Recent data show that IL4I1 can catabolize Trp to indole-3-pyruvate (I3P) (70) and that IL4I1-dependent I3P is metabolized to KynA, indole-3-aldehyde (I3A), indole-3-acetic acid (I3AA), and indole-3-lactate (I3L) in glioblastoma cells (59). Indoles derived from IL4I1-mediated Trp metabolism can activate AhR to promote cancer cell motility and suppress adaptive immunity (59). Interestingly, we find that ATP stimulation induced both IL4I1 mRNA (Fig. 6*A*) and protein expression (Fig. 6*B*) in WI-38 fibroblasts. This ATP-mediated regulation was P2Y11R-dependent, as shown by impaired ATP-mediated IL4I1 mRNA and protein upregulation when WI-38 cells were cotreated with ATP and the P2Y11R antagonist, NF-157 (Fig. 6, *A* and *B*). The P2Y11R couples mostly to G_q_, which results in PLC activation and the consequent increase of IP3 levels and calcium release. To determine whether ATP-induced IL4I1 upregulation was either upstream or downstream of PLC activation, we determined IL4I1 mRNA levels in ATP-treated WI-38 fibroblasts in the presence or absence of the PLC inhibitor U73122. We find that PLC inhibition impaired ATP-induced IL4I1 mRNA upregulation (Fig. 6*C*). We conclude that PLC activation/calcium release is upstream of IL4I1 induction by ATP.

**Figure 7:**
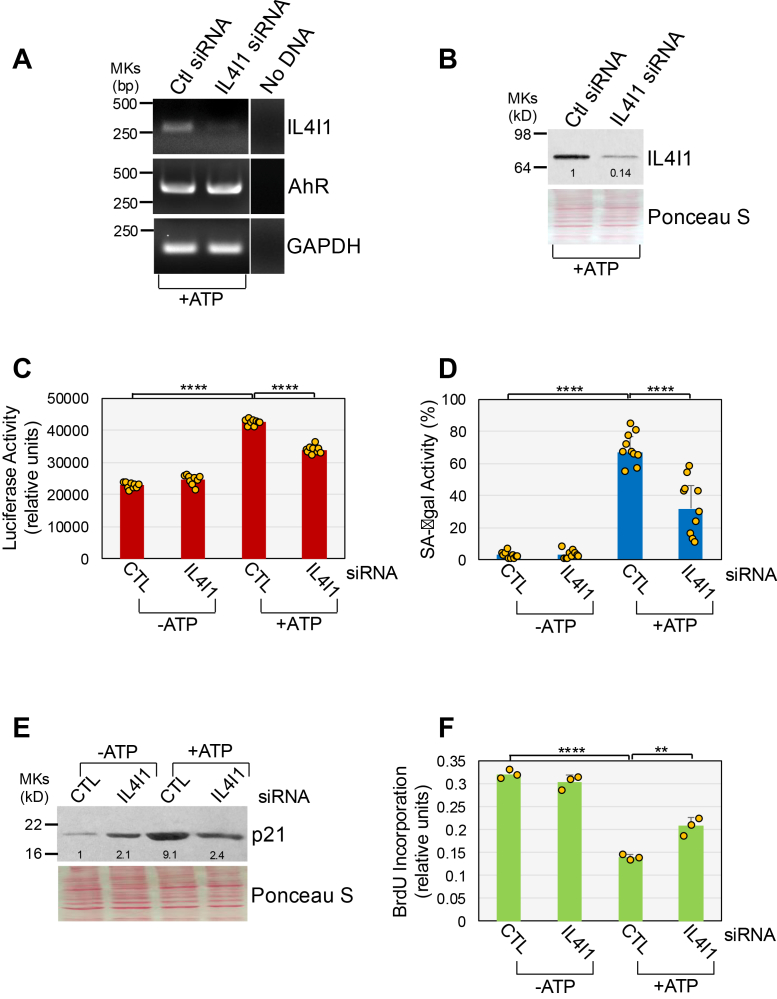
**Downregulation of IL4I1 inhibits ATP-induced AhR activation and senescence.***A* and *B*, WI-38 fibroblasts were transfected with either CTL or IL4I1 siRNA. After 24 h, cells were treated with 1.5 mM ATP for 1 day. Untreated cells served as control. IL4I1 and AhR mRNA (*A*) and IL4I1 protein (*B*) expression was quantified by RT-PCR and immunoblotting analysis, respectively. Expression of GAPDH in (*A*) served as control. Ponceau S staining in (*B*) shows equal total protein loading. Quantification of protein band intensity is shown at the bottom of the blot. *C*, WI-38 fibroblasts were transfected with either CTL or IL4I1 siRNA. After 24 h, cells were transfected with the XRE-pNL1.3 [SecNLuc] plasmid. After 24 h, cells were treated with 1.5 mM ATP for 1 day. Untreated cells served as control. Cells were collected and AhR activity was measured by luciferase assay. Values were normalized based on total protein content. *D*, *E*, and *F*, WI-38 fibroblasts were transfected with either CTL or AhR siRNA. After 24 h, cells were treated with 1.5 mM ATP for 10 days. Untreated cells served as control. In (*D*), cells were subjected to senescence-associated β-galactosidase activity staining. In (*E*), cells were collected and p21 protein expression was quantified by immunoblotting analysis using an antibody probe specific for p21. Ponceau S staining shows equal total protein loading. Quantification of protein band intensity is shown at the bottom of the blot. In (*F*), cell proliferation was quantified by BrdU incorporation assay. The Ponceau S staining shown in panels (*B*) and (*E*) is identical because both immunoblots were obtained from the same membrane following sequential probing with different antibodies. Values in *C*, *D*, and *F* represent means ± SD; statistical comparisons were made using 2-way ANOVA with *post hoc* test. ∗∗<0.01, ∗∗∗∗<0.0001. AhR, aryl hydrocarbon receptor; CTL, control; IL4I1, interleukin-4-induced-1; XRE, xenobiotic response element.

We next tested if ATP stimulation altered IL4I1-dependent Trp metabolites in human lung fibroblasts. We find that the levels of I3P-derived metabolites KynA, I3A, and I3AA were significantly increased in the culturing medium of ATP-treated WI-38 fibroblasts, as compared with culturing medium of untreated cells (Fig. 6*D*). Similarly, ATP stimulation upregulated I3L levels in the conditioned medium of WI-38 fibroblasts, although statistical significance was barely missed (Fig. 6*D*). The detection of IL4I1-dependent I3P-derived metabolites, but not of I3P itself (Fig. 6*D*), is consistent with previous reports (59, 70) and suggests that I3P is rapidly metabolized. In contrast, Kyn levels did not change in conditioned media from ATP-induced senescent cells (Fig. 6*D*). Finally, downregulation of IL4I1 mRNA by siRNA (Fig. 7*A*) suppressed IL4I1 protein expression (Fig. 7*B*) and impaired ATP-induced AhR activation (Fig. 7*C*) and premature senescence, as quantified by SA β-galactosidase activity (Fig. 7*D*), p21 protein expression (Fig. 7*E*), and cell proliferation (Fig. 7*F*). However, IL4I1 siRNA did not inhibit ATP-induced AhR mRNA upregulation (Fig. 7*A*). We confirmed these results by showing that downregulation of IL4I1 mRNA (Fig. S5*A*) and protein expression (Fig. S5*B*), using a different IL4I1-specific siRNA, inhibited ATP-induced AhR activation (Fig. S5*C*), and upregulation of SA-β-gal activity (Fig. S5*D*), and p21 expression (Fig. S5*E*). We conclude that ATP stimulation i) upregulates IL4I1 expression, and ii) promotes the accumulation of IL4I1-dependent and I3P-derived Trp metabolites, and that IL4I1 mediates ATP-induced senescence in human lung fibroblasts.

### Stimulation with IL4I1-dependent and I3P-derived Trp metabolites is sufficient to induce senescence in human lung fibroblasts

**Figure 8:**
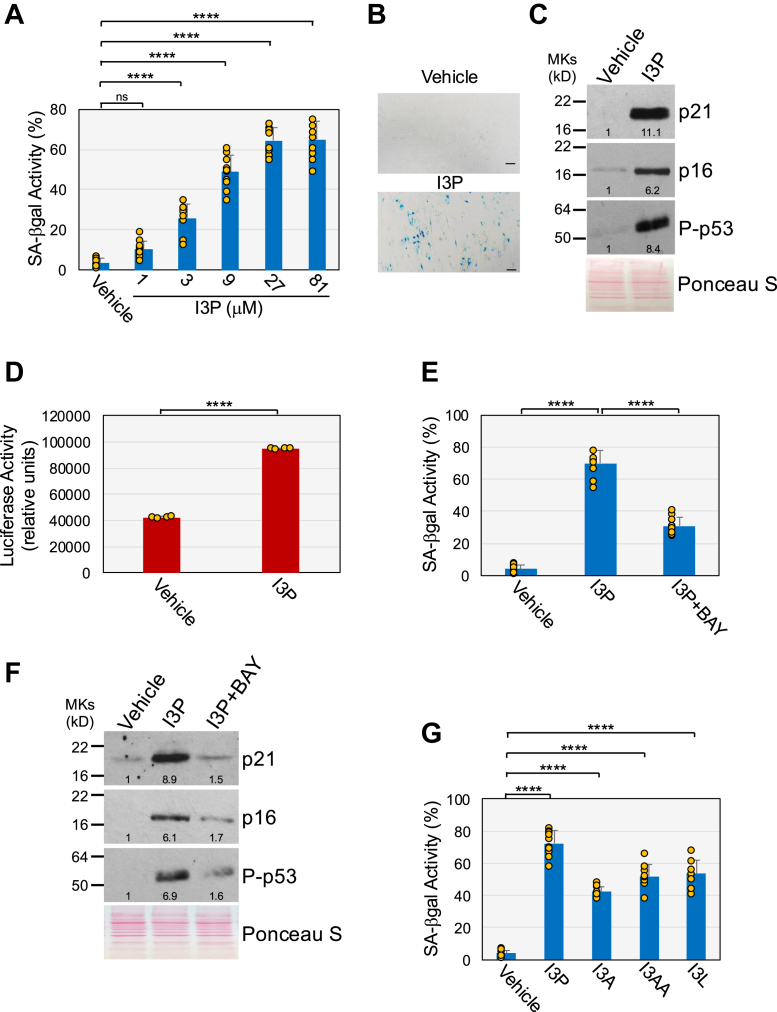
**Treatment with I3P is sufficient to activate AhR and induce premature senescence in an AhR-dependent manner.***A*, *B*, and *C*, WI-38 human diploid fibroblasts were treated with different concentrations of I3P for 10 days. In (*A* and *B*), cells were subjected to senescence-associated β-galactosidase activity staining. Quantification is shown in (*A*), representative images of cells treated with 27 μM I3P are shown in (*B*). In (*C*), cells treated with 27 μM I3P were collected and cell lysates were subjected to immunoblotting analysis using antibody probes specific for p21, p16, and phospho (Ser15)-p53. Ponceau S staining shows equal total protein loading. Quantification of protein band intensity is shown at the bottom of each blot. *D*, WI-38 fibroblasts were transfected with the XRE-pNL1.3 [SecNLuc] plasmid. After 24 h, cells were treated for 1 day with 27 μM I3P. Untreated cells served as control. Cells were collected and AhR activity measured by luciferase assay. Values were normalized based on total protein content. *E* and *F*, WI-38 cells were treated for 10 days with 27 μM I3P in the presence or absence of 10 μM BAY-218. Vehicle (DMSO)-treated cells served as control. In (*E*), cells were stained to detect senescence-associated β-galactosidase activity. In (*F*), p21, p16, and phospho (Ser15)-p53 protein expression was quantified by immunoblotting analysis using specific antibody probes. Ponceau S staining shows equal total protein loading. Quantification of protein band intensity is shown at the bottom of each blot. *G*, WI-38 fibroblasts were treated with either 27 μM I3P, I3A, I3AA, or I3L for 10 days. Untreated cells were used as control. Cells were subjected to senescence-associated β-galactosidase activity staining. Quantification is shown. Values in *A*, *D*, *E*, and *G* represent means ± SD; statistical comparisons were made using 1-way ANOVA with *post hoc* test in *A*, *E*, and *G*; statistical comparisons were made using the student’s *t* test in *D*. ∗∗∗∗<0.0001. The scale bar represents 50 μm. AhR, aryl hydrocarbon receptor; DMSO, dimethyl sulfoxide; I3P, indole-3-pyruvate; XRE, xenobiotic response element.

Since it is known that IL4I1-mediated Trp metabolism produces I3P and our data show that ATP stimulation induced senescence, upregulated IL4I1, and promoted the accumulation of IP3-derived metabolites in human fibroblasts, we asked if treatment with I3P itself is sufficient to induce senescence in human lung fibroblasts. A systematic review shows that the concentration of Trp-derived indoles in human plasma ranges from ∼1 μM to 100 μM (71). Thus, WI-38 cells were treated with I3P (1 μM to 81 μM) for 10 days in the absence of ATP stimulation. We find that treatment with I3P upregulated SA-β-gal activity in a dose-dependent manner, with 27 μM being the lowest concentration that gave the maximal effect (Fig. 8, *A* and *B*). Consistent with this result, I3P (27 μM) stimulation upregulated p21, p16, and phospho-p53 protein expression (Fig. 8*C*), and SA cell morphology (Fig. S6*A*), and inhibited cell proliferation (Fig. S6*B*). In support of I3P being downstream of Trp, treatment with I3P induced senescence in both ATP-treated and ATP-untreated WI-38 fibroblasts even when the cells were cultured in Trp-free medium (Fig. 5, *A*–*E*). We also show that I3P stimulation activated AhR (Fig. 8*D*), but did not upregulate AhR mRNA expression (Fig. S6*C*) in human lung fibroblasts. Importantly, AhR inhibition with BAY-218 impaired I3P-induced senescence in WI-38 fibroblasts, as quantified by SA-β-gal staining (Fig. 8*E*), p21, p16, and phospho-p53 protein expression (Fig. 8*F*), SA cell morphology (Fig. S6*D*), and cell proliferation (Fig. S6*E*). Neither stimulation with I3P alone nor costimulation with I3P and BAY-218 affected cell viability (Fig. S6*F*). Finally, we show that stimulation with the I3P-derived metabolites I3A, I3L, and I3AA also induced senescence of WI-38 fibroblasts (Fig. 8*G*). The senescence-inducing effect of ATP and Trp metabolites was not limited to WI-38 fibroblasts. In fact, we find that ATP stimulation upregulated IL4I1 mRNA levels (Fig. S7*A*) and induced senescence (Fig. S7*B*) in IMR-90 human fibroblasts. Moreover, we show that stimulation with either I3P or tapinarof induced senescence in IMR-90 cells (Fig. S7*B*). Together, we conclude that ATP-mediated activation of P2Y11R promotes the IL4I1-dependent conversion of Trp to I3P. Then, I3P-derived metabolites induce senescence through activation of AhR-mediated signaling in human fibroblasts.

### I3P-induced senescence in lung fibroblasts stimulates the growth of breast cancer cells

**Figure 9:**
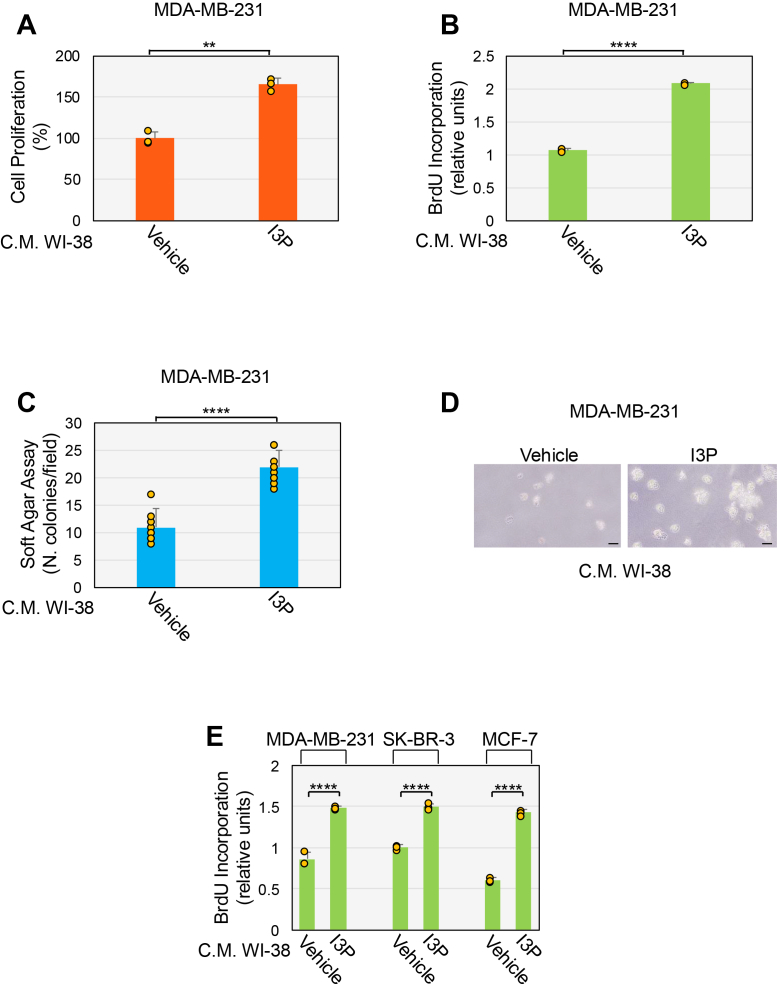
**I3P-induced senescent fibroblasts promote the growth and tumorigenic potential of breast cancer cells.***A*, *B*, *C*, and *D*, WI-38 fibroblasts were treated with 27 μM I3P for 10 days to induce senescence. Untreated cells were used as control. *A* and *B*, conditioned medium was used to culture MDA-MB-231 breast cancer cells for 48 h. Cell proliferation was quantified by both cell counting (*A*) and BrdU incorporation assay (*B*). *C* and *D*, conditioned media was used to culture MDA-MB-231 breast cancer cells for 10 days in soft agar to quantify their anchorage-independent growth. Quantification is shown in (*C*), representative images are shown in (*D*). *E*, WI-38 fibroblasts were treated with 27 μM I3P for 10 days to induce senescence. Untreated cells were used as control. Conditioned medium was used to culture MDA-MB-231, SK-BR-3, and MCF-7 breast cancer cells for 48 h. Cell proliferation was quantified by BrdU incorporation assay. Values in *A*, *B*, *C*, and *E* represent means ± SD; statistical comparisons were made using the student’s *t* test in *A*-*C*; statistical comparisons were made using 1-way ANOVA with *post hoc* test in (*E*). ∗∗<0.01, ∗∗∗∗<0.0001. The scale bar represents 50 μm. I3P, indole-3-pyruvate.

Senescent cells can release factors that promote the growth of preneoplastic and neoplastic cells, including triple-negative breast cancer (TNBC) cells (11, 12, 21, 72). Thus, we investigated whether human fibroblasts, which are induced to senesce by treatment with the Trp metabolite I3P, possess prostimulatory features toward cancer cells. More specifically, we asked if I3P-treated WI-38 fibroblasts release factors that promote the growth of the TNBC cell line MDA-MB-231. To this end, we treated WI-38 fibroblasts with I3P for 2 days, washed the cells, and cultured them for an additional 8 days to induce senescence (Fig. S7*C*) and remove any remaining I3P in the culturing media. We then used conditioned medium derived from senescent WI-38 cells to culture MDA-MB-231 cells. We find that the growth of MDA-MB-231 cells was enhanced when the cells were cultured in the presence of conditioned medium derived from I3P-treated senescent WI-38 fibroblasts, as compared to conditioned medium derived from control WI-38 cells, as shown by cell proliferation (Fig. 9*A*) and BrdU incorporation assays (Fig. 9*B*). We observed a similar increase of MDA-MB-231 cell proliferation when the cells were cultured with conditioned medium derived from I3P-treated senescent IMR-90 fibroblasts (Fig. S7*D*). Consistent with these data, we show that conditioned medium derived from WI-38 cells that were induced to senesce by I3P treatment enhanced the growth of MDA-MB-231 cells in soft agar (Fig. 9, *C* and *D*). Since it is known that AhR activation in cancer cells can have protumorigenic properties, we ruled out AhR activation in MDA-MB-231 cells when they were cultured with conditioned medium derived from WI-38 cells treated with I3P (Fig. S7*E*). As negative control, we also show that neither ATP (Fig. S7*F*) nor I3P (S7G) induced senescence in MDA-MB-231 cells. We conclude that Trp metabolite-dependent senescent lung fibroblasts release factors that promote the proliferation and anchorage-independent growth of TNBC cells, which is a key feature of cellular transformation.

Finally, we asked if the prostimulatory effect of senescent fibroblasts was limited to TNBC cells. To this end, we cultured hormone receptor-positive MCF-7 breast cancer cells and epidermal growth factor receptor 2 (HER2)-positive SK-BR-3 breast cancer cells with conditioned medium derived from I3P-treated senescent WI-38 fibroblasts. We find that, similarly to TNBC cells, senescent WI-38-derived conditioned medium increased the proliferation of both hormone receptor-positive and HER2-positive breast cancer cells (Fig. 9*E*).

## Discussion

**Figure 10:**
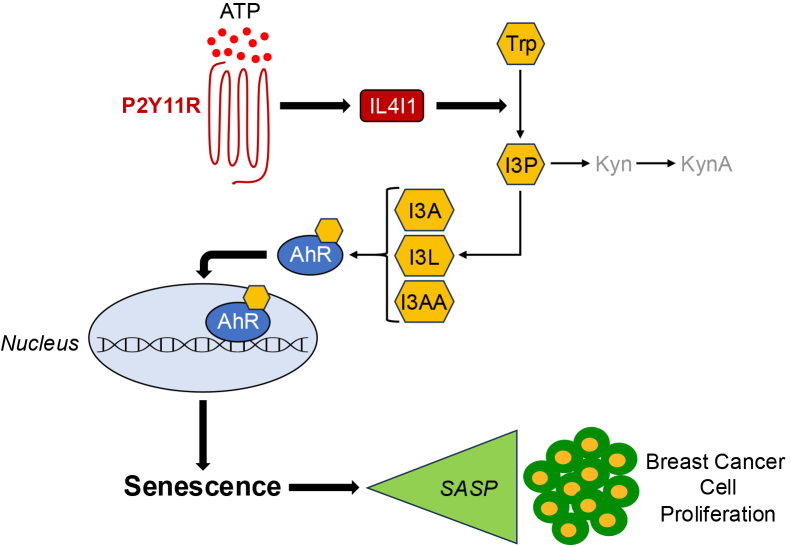
**Schematic diagram summarizing Trp metabolite-dependent senescence of human fibroblasts and their prostimulatory properties.** Activation of the P2Y11R receptor by ATP upregulates IL4I1 expression, which converts tryptophan (Trp) into I3P and its metabolites I3A, I3L, and I3AA. Trp-derived indole metabolites induce senescence by activating AhR-dependent signaling. Senescent human fibroblasts, which are induced to senescence by I3P stimulation, release SASP factors that promote the proliferation and tumorigenic potential of breast cancer cells. I3A, indole-3-aldehyde; I3AA, indole-3-acetic acid; I3L, indole-3-lactate; I3P, indole-3-pyruvate; IL4I1, interleukin-4-induced-1; P2Y11R, P2Y11 receptor; Trp, tryptophan; SASP, senescence-associated secretory phenotype.

Although it is well established that bacteria, such as those in the gut, can promote indole formation through Trp catabolism (73), the generation of Trp-derived indoles in eukaryotic cells and their functional/pathological roles are not yet well defined. In this study, we identified for the first time a novel functional link in human cells between purinergic signaling and cellular senescence that is centered on IL4I1-mediated and Trp-derived indole formation (Fig. 10). Interestingly, we find that treatment with Trp-derived indoles was sufficient to induce senescence in human lung fibroblasts. This implies that the gut microbiota has the potential to act as a double-edged sword. On the one hand, it has well established beneficial effects such as promoting gut homeostasis and regulating the immune system and metabolism. On the other hand, it might have detrimental effects by producing Trp-derived indoles, which could induce senescence either locally in the gut or at gut distal sites as indoles enter circulation.

Data show that the levels of indoles in the systemic circulation progressively decrease with aging (74, 75, 76). This is associated with the age-dependent changes in the gut composition leading to a decline in the expression and/or activity of bacterial tryptophanase (75), an enzyme that mediates Trp break down into indoles. Since senescent cells accumulate with aging, how can we reconcile our data showing that Trp-derived indoles induce senescence? We propose at least two possible explanations. First, bacteria capable of producing Trp-derived indoles can also be found in organs outside of the gut, including the lung. Thus, despite being low systemically, Trp-derived indoles might be highly concentrated locally either within the gut or at gut distal sites, where they can induce senescence of cells within their microenvironment. It would be interesting to know if bacteria producing Trp-derived indoles accumulate at sites of age-related diseases and contribute to their progression. Second, IL4I1 has been shown to promote the synthesis of I3P and its derivates (59). Here, we established for the first time that purinergic receptor stimulation induced senescence in a IL4I1/I3P-dependent manner (Fig. 10). Therefore, an alternative explanation is that host IL4I1-dependent, but microbiota-independent, Trp-derived indole formation occurs in human tissues leading to the local accumulation of senescent cells, independently of systemic indole levels.

Although there are no studies examining how IL4I1 levels change with age, the notion that IL4I1 expression is induced by cytokines and immune signaling pathway (77), and that low-grade inflammation (inflammaging) is characteristic of older age, it is plausible that IL4I1 levels could increase during aging and contribute to the accumulation of senescent cells in older individuals through Trp-derived indole synthesis. Thus, our findings pave the way for a new area of research focused on cellular senescence as a key mechanism through which conditions that promote either microbiota-mediated or host IL4I1-dependent Trp-derived indole accumulation may exert their pathological effects.

Although Trp can be metabolized to Kyn and KynA, our data show these Trp metabolites do not induce cellular senescence. In fact, inhibition of IDO1 did not inhibit ATP-induced senescence in WI-38 fibroblasts. This is in contrast with reports showing a prosenescent role of the kynurenine pathway in bone marrow cells (65, 78). We can speculate that human lung fibroblasts may be more susceptible to senescence when exposed to I3P-derived indoles compared to Kyn. An alternative explanation is that purinergic receptor activation favors the conversion of Trp to I3P-derived indoles through IL4I1, at the expense of IDO1-mediated Kyn synthesis.

What is downstream of Trp-derived indole formation that mediates ATP-induced senescence? Trp metabolites can activate AhR. We find that ATP stimulation upregulated and activated AhR in a P2Y11R-dependent manner, that AhR activation was required for both ATP- and I3P-induced senescence, and that AhR stimulation *per se* was sufficient to induce senescence in human fibroblasts. Interestingly, we also find that IL4I1 knockdown did not inhibit ATP-induced AhR mRNA upregulation and that I3P treatment did not upregulate AhR mRNA levels. Together, these data suggest that ATP/IL4I1/I3P-dependent signaling mostly promotes AhR activation, which is reinforced by ATP-dependent, but I3P-independent, AhR mRNA upregulation. These data support previous reports showing that AhR activation can promote senescence by inhibiting cell proliferation, upregulating p16 and p21 expression, inducing mitochondrial dysfunction, and promoting oxidative stress (65, 66, 67). In contrast, mouse embryonic fibroblasts derived from AhR null mice showed earlier senescence, as compared to their WT counterpart, during adipogenic differentiation (79), and AhR null mice displayed increased age-induced senescence in the liver (80). Thus, the role of AhR in senescence could be dependent on the cell type and the ligand through which AhR gets activated. Nevertheless, our data link for the first time Trp metabolism to AhR activation in the context of cellular senescence induced by purinergic signaling activation in human cells.

We have previously shown that P2Y11R activation leads to cellular senescence through mitochondrial ROS generation following PLC-dependent calcium release from the endoplasmic reticulum and mitochondrial calcium overload (50). Oxidants can activate the DNA damage response resulting in p53 activation and p21 expression (81). Mitochondrial-derived oxidants can also act as signaling molecules and regulate gene transcription. Since we find in this study that PLC activation was upstream of IL4I1 mRNA upregulation following ATP stimulation, downregulation of IL4I1 impaired ATP-induced AhR activation, and AhR inhibition/downregulation inhibited ATP-induced p53 phosphorylation, and p21 upregulation, we can speculate that P2Y11R activation by ATP promotes p21 upregulation and senescence in a manner that is dependent on both activation of a DNA damage response and IL4I1/I3P-mediated and AhR-dependent gene transcription following mitochondrial-derived ROS generation.

Data show that purinergic signaling controls the host–tumor interaction by modulating both cancer and immune cells. It is known that ATP levels are higher in the TME, as compared to healthy tissues (68, 82, 83). In the TME, ATP promotes cancer cell growth, survival and metastatic potential through P2R-mediated signaling within the cancer cells (84, 85, 86, 87, 88). However, if purinergic signaling within the fibroblastic cell component of the TME controls tumor progression remains largely unknown. We find that conditioned medium derived from senescent fibroblasts, which were induced to senesce by I3P stimulation, enhanced both the proliferation of MDA-MB-231 TNBC cells and their anchorage-independent growth in soft agar. These results are consistent with our previously published data showing that conditioned medium from ATP-stimulated senescent WI-38 fibroblasts enhanced proliferation and growth in soft agar of MDA-MB-231 cells, in an amphiregulin-dependent manner (50). Interestingly, in our current studies, we find that amphiregulin was a SASP factor that, together with IL-8 and CXCL2, was upregulated by ATP stimulation in an AhR-dependent manner. We speculate that amphiregulin may contribute to the prostimulatory abilities of the conditioned medium derived from senescent lung fibroblasts following I3P stimulation. We also found that the prostimulatory properties of the ATP-dependent SASP in human fibroblasts were not only limited to triple negative breast cancer cells, but also extended to hormone receptor-positive and HER2-positive breast cancer cell lines. Together, our findings indicate that Trp-derived indoles indirectly enhance breast cancer cell proliferation by inducing senescence of lung fibroblasts (Fig. 10).

Interestingly, IL4I1 expression is increased in several cancers, including invasive breast cancer (89). IL4I1 has also been associated with reduced survival in glioma patients (59). In addition, by promoting cancer cell motility and suppressing adaptive immunity, IL4I1 enhances the progression of chronic lymphocytic leukemia in mice (59). Thus, given the known protumorigenic role of senescent fibroblasts, the ability of IL4I1 to mediate the synthesis of Trp-derived indoles, and our data showing that Trp-derived indoles can promote senescence, our findings (i) broadly support a protumorigenic role for IL4I1, and (ii) specifically suggest a novel tumor-promoting function of IL4I1 within the TME through its ability to induce senescence of cancer-associated fibroblasts *via* a mechanism that relies on Trp-derived metabolites.

Breast cancer cells metastasize to the lungs. WI-38 and IMR-90 fibroblasts are of lung origin. Thus, we can speculate that senescent fibroblasts may create a permissible niche within the lungs, in a purinergic/indole-dependent manner, which fuels breast cancer cell growth. Moreover, our studies have a potential translational impact in the field of cancer: we envision prevention of premature senescence of fibroblasts within the TME, using either P2Y11R or AhR inhibitors, as possible alternative therapeutic options aimed at blocking or limiting the growth of breast cancer cells at metastatic sites.

## Experimental procedures

### Materials

Antibodies were obtained from the following sources: anti-p21 (F-5; 1:1000 dilution) IgGs were from Santa Cruz Biotechnology; anti-phospho-p53 (Ser15) (pAb9284; 1:1000 dilution) IgGs were from Cell Signaling; anti-p16 (mAb EPR20418; 1:500 dilution) and anti-AhR (ab190797; 1:500 dilution) IgGs were from Abcam; anti-IL4I1 (NBP3-03586; 1:500 dilution) IgGs were from Novus Biologicals. ATP was from Sigma-Aldrich. I3P, I3A, I3L, and I3AA were from Cayman Chemical. Tapinarof, a selective AhR agonist, was from MedChemExpress LLC. Silencer Select negative control siRNA (4390843), Silencer Select AhR siRNA (s1200), Silencer Select AhR siRNA#2 (s1199), Silencer Select IL4I1 siRNA (s48970), and Silencer Select IL4I1 siRNA#2 (s48971) were from Thermo Fisher Scientific. All other biochemical reagents were of the highest available purity and were commercially obtained.

### Pharmacological inhibitors and antagonists

U73122, a PLC inhibitor (90), NF157, a selective P2Y11R antagonist (91), and BAPTA-AM, a highly selective intracellular calcium chelator (92), were from Tocris Bioscience. BAY-218, a selective AhR inhibitor (93), was from Sigma-Aldrich. Epacadostat, a selective IDO1 inhibitor (94, 95), was from Selleck Chemicals LLC.

### Cell culture

WI-38 and IMR-90 human lung fibroblasts were purchased from American Type Culture Collection (ATCC) and cultured in Eagle’s Minimal Essential Medium supplemented with 2 mM glutamine, 100 U/ml penicillin, 100 μg/ml streptomycin, and 10% fetal bovine serum (FBS). For Trp-depletion experiments, WI-38 cells were cultured in Trp-free Dulbecco’s Minimal Essential Medium (US Biologicals) supplemented with 2 mM glutamine, 100 U/ml penicillin, 100 μg/ml streptomycin, and dialyzed FBS (Thermo Fisher Scientific Inc.). All experiments with WI-38 and IMR-90 fibroblasts were performed with cells at population doubling between 10 and 20. MDA-MB-231 cells were cultured in Dulbecco’s Minimal Essential Medium supplemented with 2 mM glutamine, 100 U/ml penicillin, 100 μg/ml streptomycin, and 10% FBS. MCF-7 were purchased from ATCC and cultured in Eagle’s Minimal Essential Medium supplemented with 2 mM glutamine, 100 U/ml penicillin, 100 μg/ml streptomycin, 10% FBS, 1% nonessential amino acids, and 0.01 mg/ml human recombinant insulin. SK-BR-3 cells were provided by Dr Steffi Oesterreich (University of Pittsburgh) and cultured in McCoy’s 5A Medium supplemented with 2 mM glutamine, 100 U/ml penicillin, 100 μg/ml streptomycin, and 10% FBS.

### Luciferase reporter assay

Cells were seeded in 60-mm dishes at 270,000 per dish. The following day, cells were transiently transfected, using Lipofectamine 3000 Transfection Reagent (catalog number L3000008, Thermo Fisher Scientific Inc.), with 10 μg of xenobiotic response element -pNL1.3 [SecNLuc] construct (catalog number 182295; Addgene) (96). Twenty-four hours posttransfection, cells were split into 6-well plates and treated as described in the results section. After 24 h, 100 μl of supernatant was used to quantify secreted NanoLuc luciferase using the Nano-Glo Luciferase Assay System (catalog number N1110, Promega) (97). Results were normalized based on protein concentration, as determined using the BCA Assay kit from Sigma-Aldrich.

### Immunoblotting

Cells were collected in boiling Laemmli buffer (62.5 mM Tris-HCl pH 6.8, 2% SDS, 10% glycerol, 5% β-mercaptoethanol, and 0.01% bromophenol blue). Cellular proteins were resolved by SDS-PAGE (12.5% acrylamide) and transferred to Amersham Protran 0.2 μm nitrocellulose blotting membrane (GE Healthcare Life Sciences). Blots were incubated for 1 h and 15 min in tris-buffered saline with tween 20 (TBST) (10 mM Tris-HCl, pH 8.0, 150 mM NaCl, and 0.2% Tween 20) containing 2% powdered skim milk and 1% bovine serum albumin. After three washes with TBST, membranes were incubated overnight with the primary antibody and washed three more times with TBST. Blots were then incubated for 1 h and 15 min with horseradish peroxidase-conjugated goat anti-rabbit/mouse IgG. Bound proteins were detected using an ECL detection kit (Pierce) according to the manufacturer’s protocol. Representative images are shown. *Quantification of protein expression*: sample protein concentration was determined using the BCA Assay kit from Sigma-Aldrich. Equal total proteins were resolved by SDS-PAGE. Equal protein loading was assessed by Ponceau S staining. Bands were scanned and their density was quantified using Image J and normalized based on Ponceau S intensity.

### SA-β-gal activity assay

SA-β-gal activity was measured using the Senescence β-Galactosidase Staining Kit according to the manufacturer’s protocol (catalog number 9860, Cell Signaling Technology) (1). Average percent senescence was calculated from quantification of total cells and senescent cells in 10 fields of view per condition, using an inverted Olympus microscope (CKX53). Representative images of microscope fields are shown.

### Senescence-associated cell morphology

The percentage of cells displaying a flat and large cell morphology was calculated from 10 fields of view per condition using an inverted Olympus microscope (CKX53). Representative images of microscope fields are shown.

### Transfection of siRNA

siRNA was introduced into cells using Lipofectamine RNAiMax Reagent (catalog number 13778-030, Life Technologies Frederick) according to the manufacturer’s protocol, using 40 pmol per well of 6-well culture plates.

### RNA isolation and RT-PCR

Cells were collected and total RNA was isolated using the RNeasy Mini kit (catalog number 74134, QIAGEN,). Equal amounts of RNA were treated with RNase-free DNase and subjected to reverse transcription using the Advantage RT-for-PCR kit (catalog number 639505, Clontech Laboratories, Inc.), according to the manufacturer’s protocol (98). PCR was then performed for each gene studied in its linear zone of amplification using the following primers: hAhR forward-aatgtacagagctggactacc; hAhR reverse-acggatgatgaagtggctgaa (25 cycles); hIL4I1 forward-cgcccgaagacatctaccaga; hIL4I1 reverse-tactggagtctgtcgctgagg (30 cycles); hIL-8 forward-gttttgccaaggagtgctaaa; hIL-8 reverse-ctccacaaccctctgcaccca (25 cycles); hCXCL2 forward-cagcaggagcgcccctggcca; hCXCL2 reverse-tgccatttttcagcatctttt (30 cycles); hAmphiregulin forward-tgcattcacggagaatgcaaa; hAmphiregulin reverse-tcctcagcttctccttcatat (25 cycles); GAPDH forward-gcaaattccatggcaccgt; GAPDH reverse-tcgccccacttgattttgg (25 cycles). PCR reactions were performed in a final volume of 50 μl containing 1 × PCR buffer (Roche), 200 μM each deoxyribonucleotide triphosphate, 0.5 μM of each forward and reverse primer, 1 U Taq DNA polymerase (Roche), and template DNA (100 ng). Nuclease-free water was added to reach the final reaction volume. Amplifications were carried out in a thermal cycler with the following conditions: an initial denaturation step at 95 °C for 5 min, followed by 25 to 30 cycles of denaturation at 95 °C for 30 s, primer annealing at 55 to 65 °C for 30 s (depending on primer melting temperature), and extension at 72 °C for 1 min per kb of target sequence. A final extension step was performed at 72 °C for 10 min. PCR products were subsequently analyzed by agarose gel electrophoresis.

### BrdU incorporation assay

Cell proliferation was measured and quantified using the Cell Proliferation ELISA, BrdU (colorimetric) kit (catalog number 11647229001, Roche,) (99). The kit was used according to the manufacturer’s protocol. Cells were incubated with BrdU labeling solution overnight at 37 °C. The absorbances were measured at 370 nm following substrate incubation using an ELISA plate reader.

### High-resolution LC-HRMS

#### Sample preparation

Metabolic quenching and polar metabolite pool extraction was performed by adding ice cold 1:1 methanol:ethanol at a ratio of 1:4 cell supernatant. Deuterated (D_3_)-creatinine and (D_3_)-alanine, (D_4_)-taurine and (D_3_)-lactate (Sigma-Aldrich) was added to the sample lysates as an internal standard for a final concentration of 10 μM. After 3 min of vortexing, the supernatant was cleared of protein by centrifugation at 16,000*g* for 5 min 400 μl cleared supernatant was dried to completion under N_2_ gas and resuspended in 40 μl water/0.1% formic acid. 2 μl of cleared supernatant was subjected to online LC-MS analysis.

#### LC-HRMS method

Analyses were performed by untargeted LC-HRMS. Briefly, samples were injected *via* a Thermo Vanquish UHPLC and separated over a reversed phase Phenomenex Kinetix C18 column (2.1 × 150 mm, 1.7 μm particle size) maintained at 55 °C. For the 8 min LC gradient, the mobile phase consisted of the following: solvent A (water/5 mM ammonium acetate) and solvent B (methanol). The gradient was the following: 0.3 min 5% B, increase to 30%B over 0.5 min, continue increasing to 60%B over 1 min, hold at 60%B for 0.7 min, increasing to 98%B over 0.5 min, hold at 98%B for 2.5 min, and re-equilibrate at 5%B for 4 min. The Thermo IDX tribrid mass spectrometer was operated in polarity switching mode, scanning both positive and negative ion mode, in ddMS^2^ mode (2 μscans) from 70 to 800 m/z at 120,000 resolution with an AGC target of 2e5 for full scan, 2e4 for ms^2^ scans using HCD fragmentation at stepped 15, 35, 50 collision energies. Source ionization setting was 3.0 and 2.4 kV spray voltage respectively for positive and negative mode. Source gas parameters were 35 sheath gas, 12 auxiliary gas at 320 °C, and 8 sweep gas. Calibration was performed prior to analysis using the Pierce FlexMix Ion Calibration Solutions (Thermo Fisher Scientific). Integrated peak areas were then extracted manually using Quan Browser (Thermo Fisher Xcalibur ver. 2.7) and normalized to internal standard peak areas.

### 3-[4,5-dimethylthiazol-2-yl]-2,5-diphenyl tetrazolium bromide assay

The 3-[4,5-dimethylthiazol-2-yl]-2,5-diphenyl tetrazolium bromide assay kit (M6494) was purchased from Thermo Fisher Scientific and the assay was performed according to the manufacturer’s recommendations (100).

### Growth in soft agar

Cells (5 × 10^4^) were suspended in 3 ml of complete medium and 0.33% SeaPlaque low-melting temperature agarose. These cells were plated over a 2-ml layer of solidified complete medium and 0.5% agarose and allowed to settle to the interface between these layers at 37 °C. After 30 min, the plates were allowed to harden at room temperature for 30 min before returning to 37 °C. After 10 days, colonies were photographed under low magnification. The colonies in 60 randomly chosen fields from three independent plates were counted.

### Statistical analysis

Studies were performed in triplicate using three biological replicates to achieve statistically significant differences. The mean ± SD is shown. Significance was calculated using either the Student’s *t* test when comparing two data sets, or 1-way/2-way ANOVA followed by Tukey’s *post hoc* test, when comparing more than two data sets.

## Data availability

All data are contained within the article.

## Supporting information

This article contains supporting information.

## Conflict of interest

The authors declare that they have no conflicts of interest with the contents of this article.
