# Supplementary material for: Extracellular ATP drives tryptophan metabolism and aryl hydrocarbon receptor activation to promote cellular senescence
Source: J Biol Chem. 2026 Apr 27;302(6):113094. doi: 10.1016/j.jbc.2026.113094 (PMC13234232; doi:10.1016/j.jbc.2026.113094)
Supplement: Supplementary Figures [file mmc1.pdf]

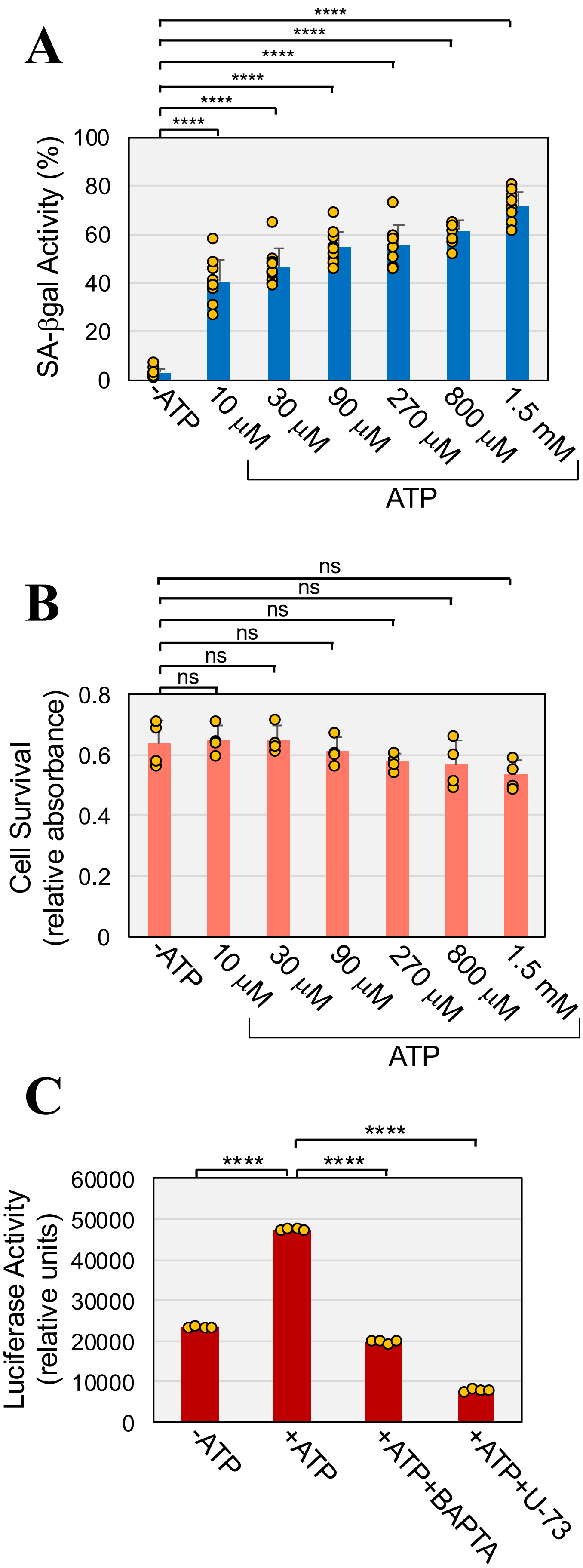
A
****
****
****
****
****
****
100
80
60
40
20
0
SA-βgal Activity (%)
-ATP
10 μM
30 μM
90 μM
270 μM
800 μM
1.5 mM
ATP
B
ns
ns
ns
ns
ns
ns
0.8
0.6
0.4
0.2
0
Cell Survival
(relative absorbance)
-ATP
10 μM
30 μM
90 μM
270 μM
800 μM
1.5 mM
ATP
C
****
****
****
60000
50000
40000
30000
20000
10000
0
Luciferase Activity
(relative units)
-ATP
+ATP
+ATP+BAPTA
+ATP+U-73

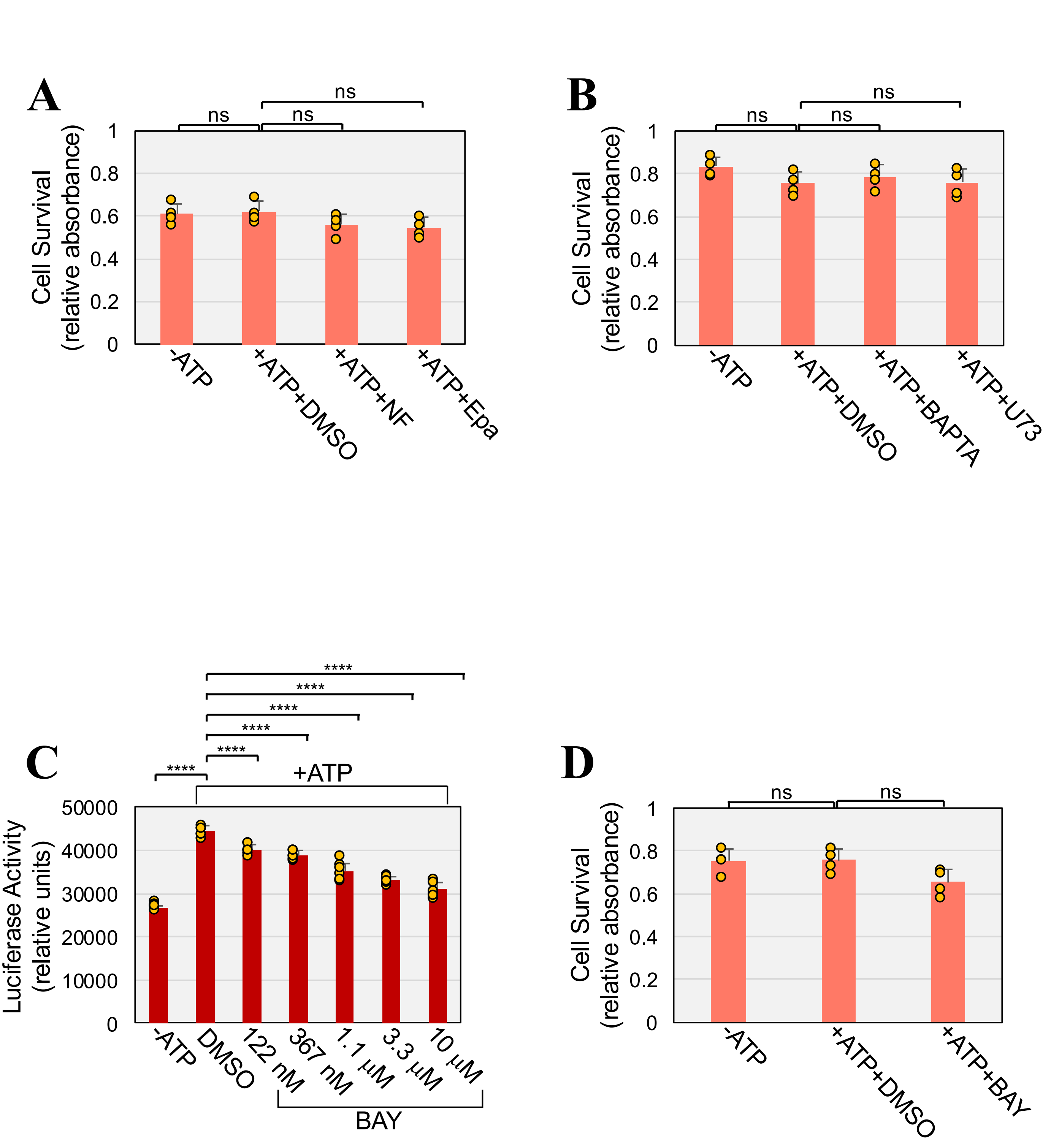
A
ns
ns
ns
Cell Survival
(relative absorbance)
1
0.8
0.6
0.4
0.2
0
-ATP
+ATP+DMSO
+ATP+NF
+ATP+Epa
B
ns
ns
ns
Cell Survival
(relative absorbance)
-ATP
+ATP+DMSO
+ATP+BAPTA
+ATP+U73
C
****
+ATP
Luciferase Activity
(relative units)
50000
40000
30000
20000
10000
0
-ATP
DMSO
122 nM
367 nM
1.1 µM
3.3 µM
10 µM
BAY
D
ns
ns
Cell Survival
(relative absorbance)
-ATP
+ATP+DMSO
+ATP+BAY

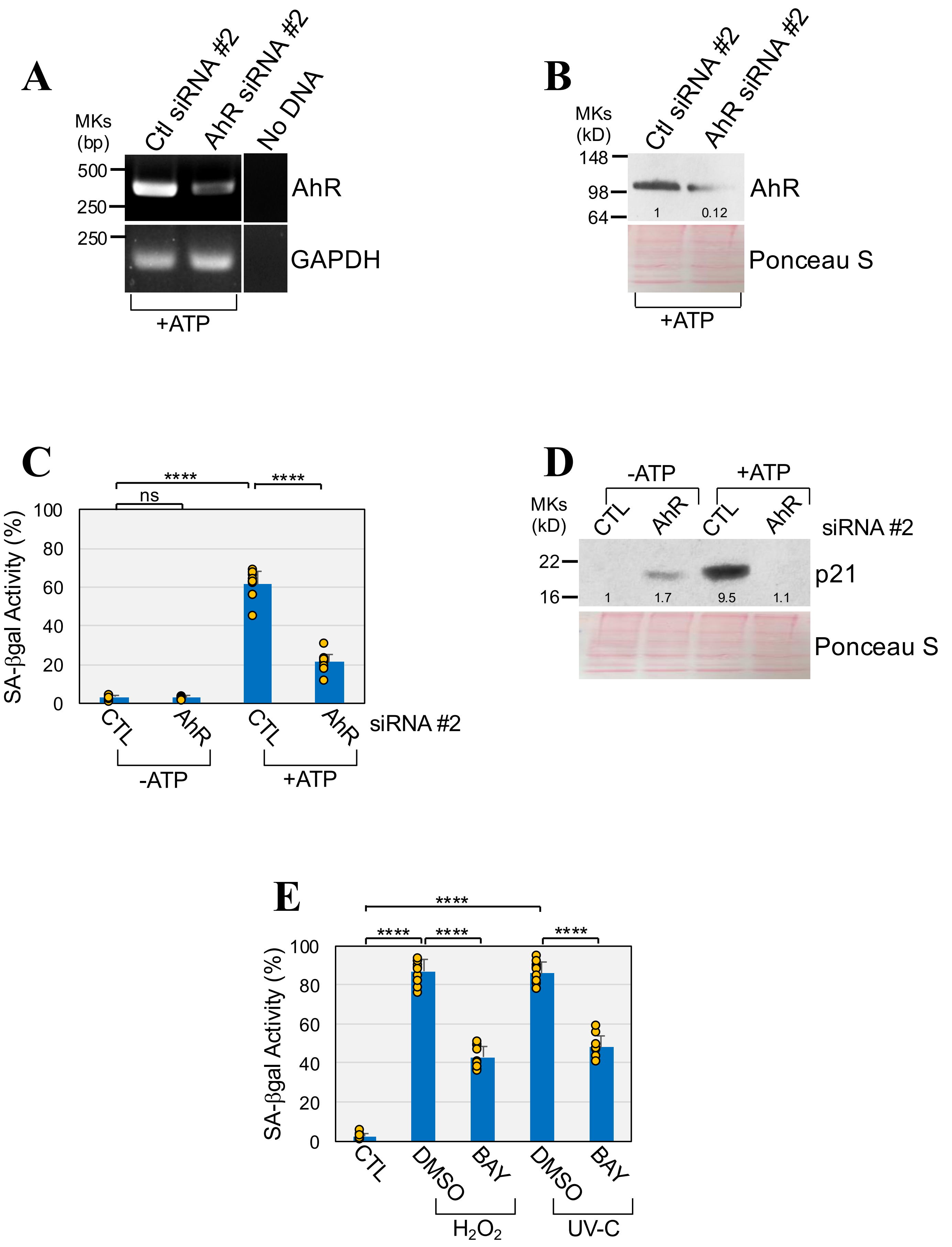
A
MKs (bp)
Ctl siRNA #2
AhR siRNA #2
No DNA
500
250
AhR
250
GAPDH
+ATP
B
MKs (kD)
Ctl siRNA #2
AhR siRNA #2
148
98
64
AhR
1
0.12
Ponceau S
+ATP
C
SA-βgal Activity (%)
100
80
60
40
20
0
ns
****
****
CTL
AhR
CTL
AhR
siRNA #2
-ATP
+ATP
D
-ATP
+ATP
MKs (kD)
CTL
AhR
CTL
AhR
siRNA #2
22
16
p21
1
1.7
9.5
1.1
Ponceau S
E
SA-βgal Activity (%)
100
80
60
40
20
0
****
****
****
****
CTL
DMSO
BAY
DMSO
BAY
H2O2
UV-C

Volonte et al, Fig S4

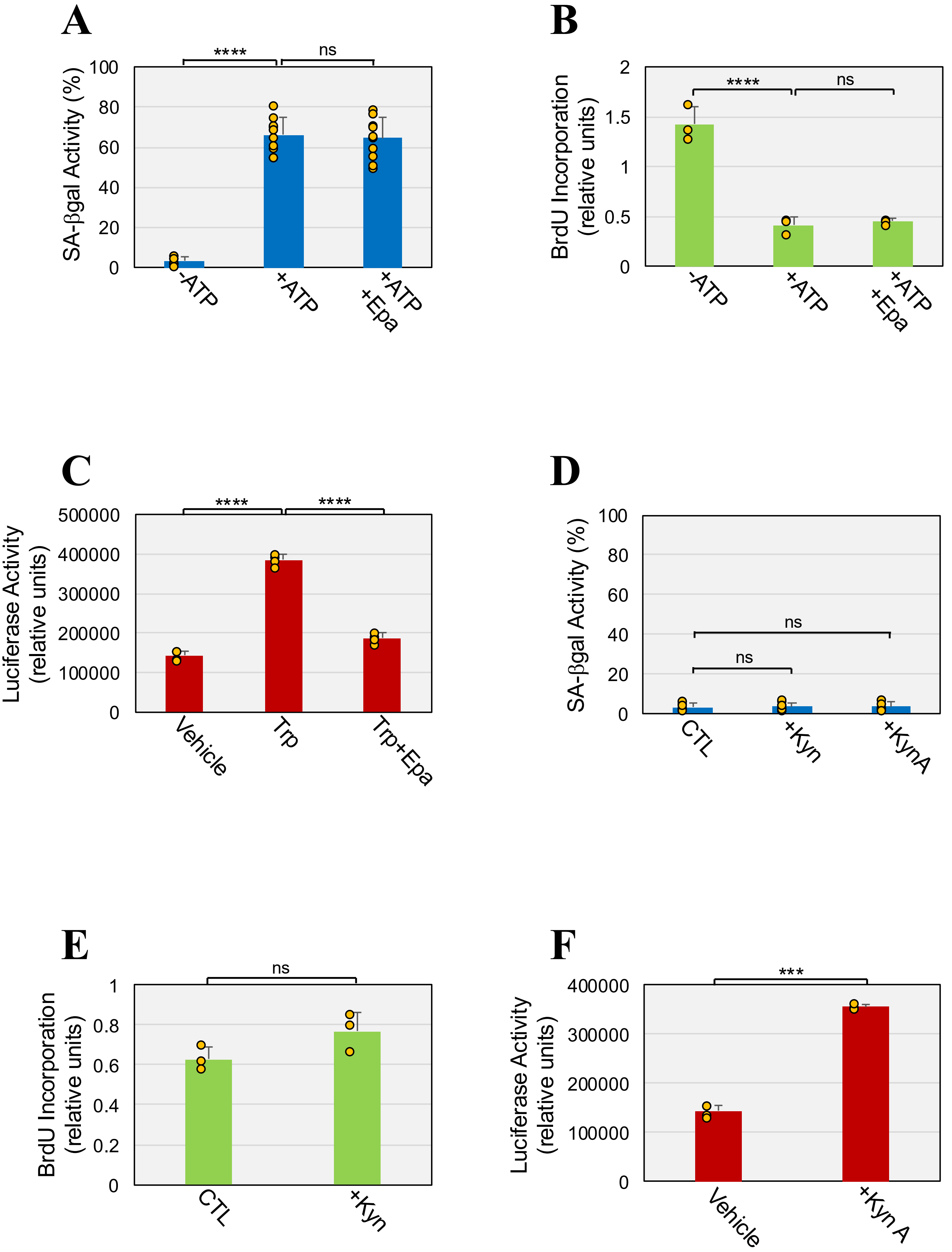

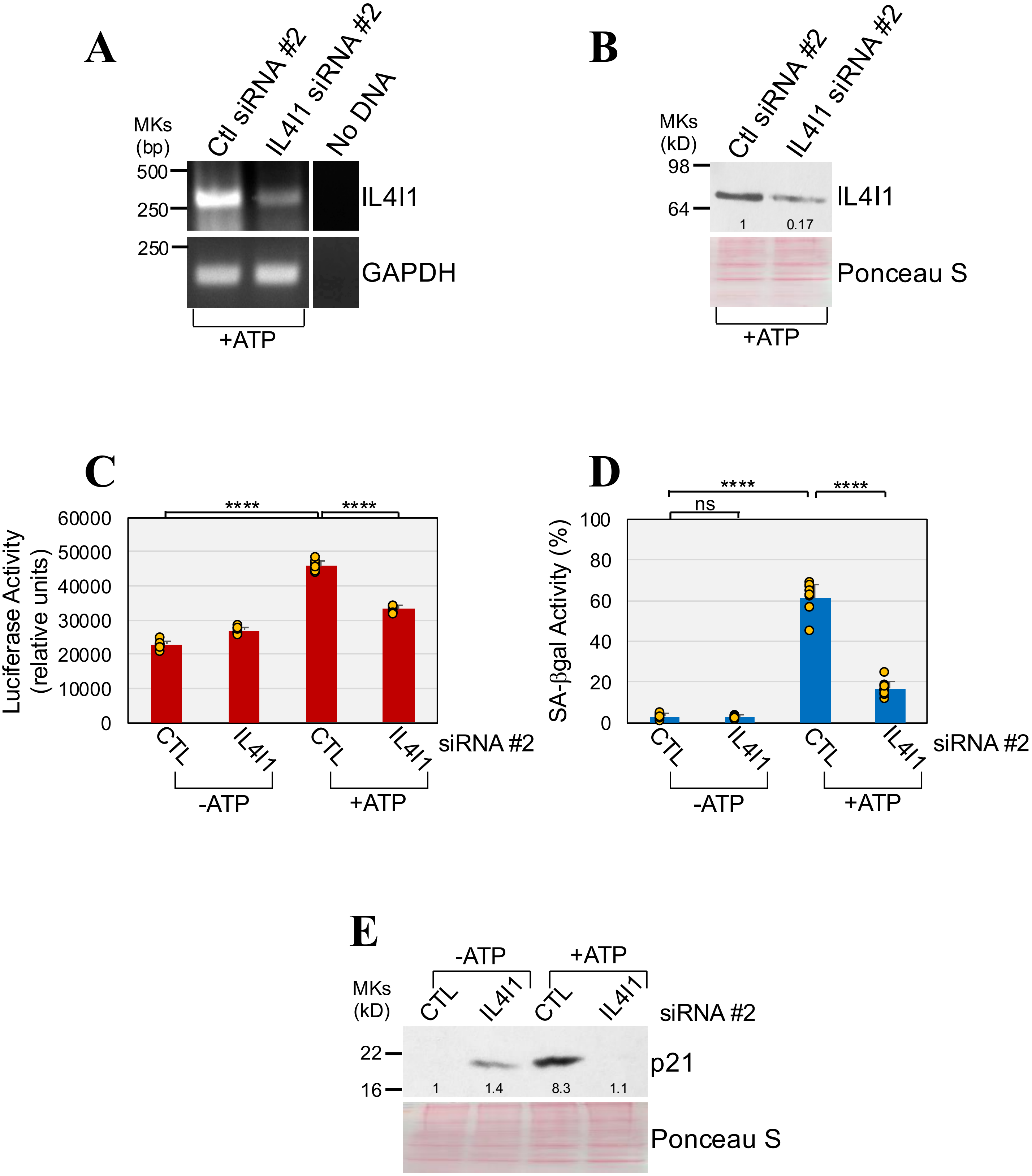
A
MKs
(bp)
Ctl siRNA #2
IL4I1 siRNA #2
No DNA
500
250
IL4I1
250
GAPDH
+ATP
B
MKs
(kD)
Ctl siRNA #2
IL4I1 siRNA #2
98
64
IL4I1
1
0.17
Ponceau S
+ATP
C
Luciferase Activity
(relative units)
60000
50000
40000
30000
20000
10000
0
****
****
CTL
IL4I1
CTL
IL4I1
siRNA #2
-ATP
+ATP
D
SA-βgal Activity (%)
100
80
60
40
20
0
****
ns
****
CTL
IL4I1
CTL
IL4I1
siRNA #2
-ATP
+ATP
E
-ATP
+ATP
MKs
(kD)
CTL
IL4I1
CTL
IL4I1
siRNA #2
22
16
p21
1
1.4
8.3
1.1
Ponceau S

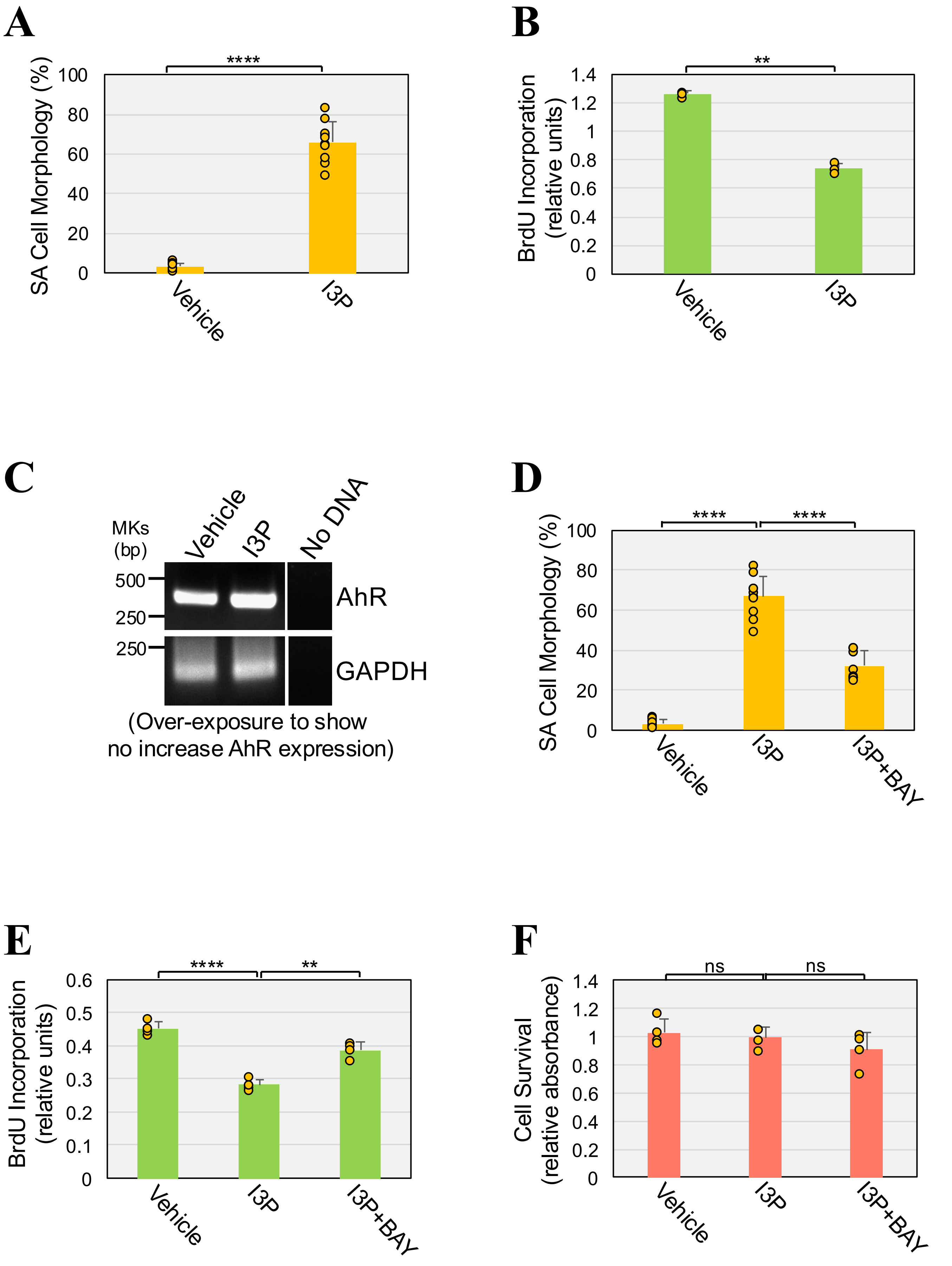
A
****
SA Cell Morphology (%)
100
80
60
40
20
0
Vehicle
I3P
B
**
BrdU Incorporation (relative units)
1.4
1.2
1
0.8
0.6
0.4
0.2
0
Vehicle
I3P
C
MKs (bp)
Vehicle
I3P
No DNA
500
250
AhR
250
GAPDH
(Over-exposure to show no increase AhR expression)
D
****
****
SA Cell Morphology (%)
100
80
60
40
20
0
Vehicle
I3P
I3P+BAY
E
****
**
BrdU Incorporation (relative units)
0.6
0.5
0.4
0.3
0.2
0.1
0
Vehicle
I3P
I3P+BAY
F
ns
ns
Cell Survival (relative absorbance)
1.4
1.2
1
0.8
0.6
0.4
0.2
0
Vehicle
I3P
I3P+BAY

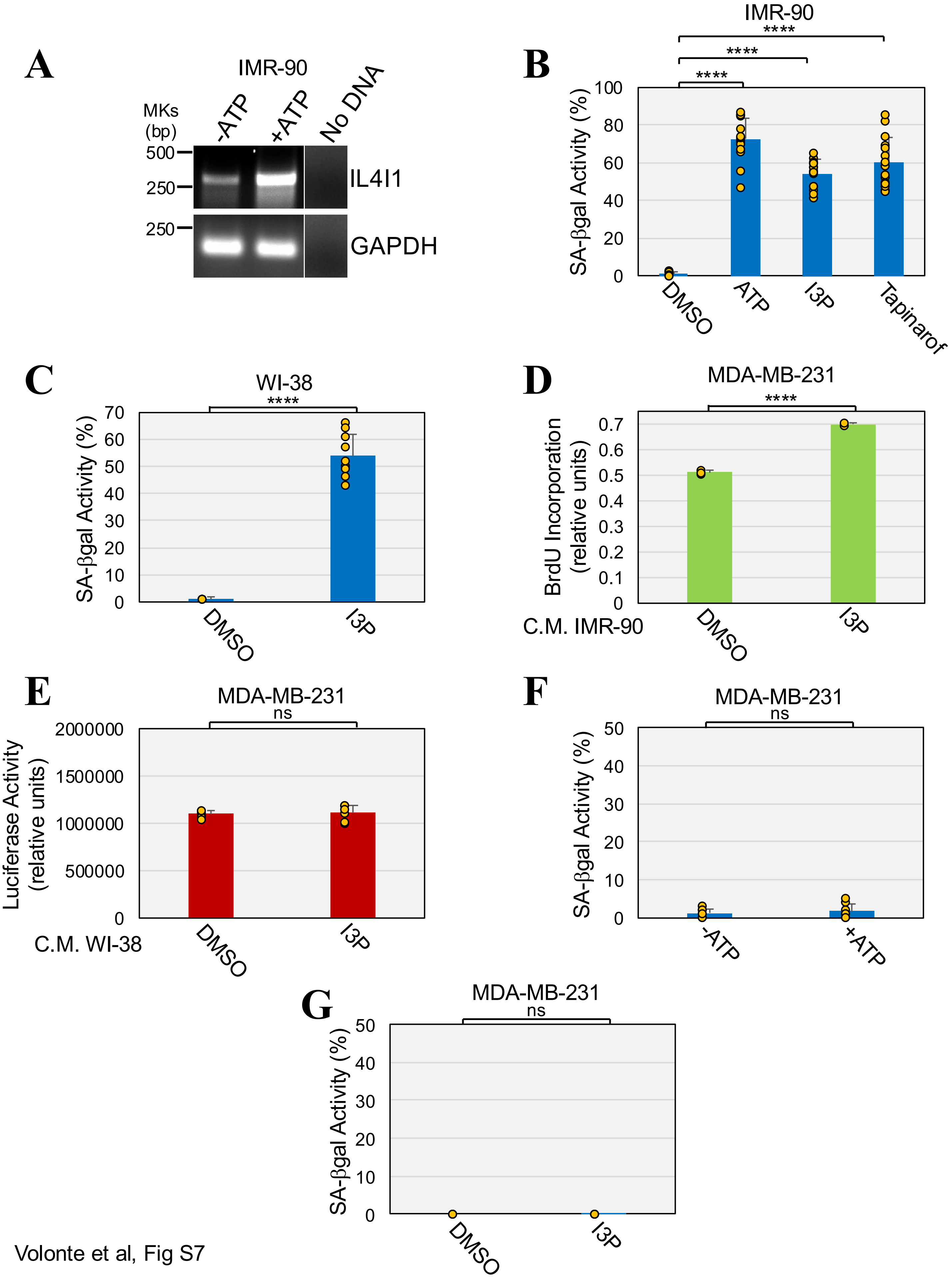

Volonte et al, Fig S7

SUPPLEMENTAL FIGURE LEGENDS

Figure S1. ATP induces senescence without affecting cell viability. ATP activates AhR in a PLC- and calcium-dependent manner. *(A,B)* WI-38 fibroblasts were treated with different ATP concentrations (10 μM to 1.5 mM) for 10 days. Untreated cells served as control. In (*A*), senescence was quantified by senescence-associated β-galactosidase activity. In (*B*), cell viability was quantified by MTT assay. (*C*) WI-38 fibroblasts were transfected with the XRE-pNL1.3 [SecNLuc] plasmid. After 24 hours, cells were treated for 1 day with 1.5 mM ATP in the presence or absence of either BAPTA-AM (5 μM) or U-73121 (U73; 3 μM). Untreated cells served as control. Cells were collected and AhR activity was measured by luciferase assay. Values were normalized based on total protein content. Values represent means ± St Dev; statistical comparisons were made using 1-way ANOVA with post hoc test. ****<0.0001.

Figure S2. Cell survival analysis following pharmacological interventions. Dose-response inhibition of AhR activation by BAY-218. WI-38 fibroblasts were treated with 1.5 mM ATP for 1 day in the presence or absence of either NF-157 (40 μM) (*A*), Epacadostat (15 μM) (*A*), BAPTA-AM (5 μM) (*B*) or U-73121 (3 μM) (*B*). Cell viability was quantified by MTT assay. (*C*) WI-38 fibroblasts were transfected with the XRE-pNL1.3 [SecNLuc] plasmid. After 24 hours, cells were treated for 1 day with 1.5 mM ATP in the presence or absence of different BAY-218 concentrations (122 nM to 10 μM). Untreated cells served as control. Cells were collected and AhR activity was measured by luciferase assay. Values were normalized based on total protein content. (*D*) WI-38 fibroblasts were treated with 1.5 mM ATP for 10 days in the presence or absence of BAY-218 (10 μM). Cell viability was quantified by MTT assay. Values represent means ± St Dev; statistical comparisons were made using 1-way ANOVA with post hoc test. ****<0.0001.

Figure S3. Downregulation of AhR inhibits ATP-induced senescence. Inhibition of oxidative stress- and UV-C-induced senescence by BAY-218. (*A-D*) WI-38 fibroblasts were transfected with either CTL or AhR siRNA #2. After 24 hours, cells were treated with 1.5 mM ATP for either 1 (*A,B*) or 10 (*C,D*) days. Untreated cells served as control. In *(A),* AhR mRNA expression was quantified by RT-PCR analysis using AhR-specific primers. GAPDH expression served as control. In (*B*), AhR protein level was determined by immunoblotting analysis. Ponceau S staining shows equal total protein loading. Quantification of protein band intensity is shown at the bottom of the blot. In *(C),* cellular senescence was quantified by senescence-associated β-galactosidase activity staining. In (*D*), p21 protein expression was quantified by immunoblotting analysis. Ponceau S staining shows equal total protein loading. Quantification of protein band intensity is shown at the bottom of the blot. (*E*) WI-38 cells were treated with sublethal doses of either hydrogen peroxide (H_2O_2; 450 μM for 2 hours) or UV-C light (10 J/m^2). Cells were recovered in complete medium for 10 days in the presence or absence of 10 μM BAY-218. Untreated cells were used as control. Senescence was quantified by senescence-associated β-galactosidase activity. The Ponceau S staining is reused between panels *(B)* and *(D)*, reflecting that both immunoblots were performed on the same membrane with sequential antibody probing. Values in C and E represent means ± St Dev; statistical comparisons were made using 2-way ANOVA with post hoc test. ****<0.0001.

Figure S4. Inhibition of IDO1 does not prevent ATP-induced senescence. Kynurenin stimulation does not promote senescence. *(A,B)* WI-38 fibroblasts were treated with 1.5 mM

ATP for 10 days in the presence or absence of either DMSO or Epacadostat (15 μM). Untreated cells were used as control. In *(A)*, cells were stained to detect senescence-associated β-galactosidase activity. Quantification is shown. In *(B)*, BrdU incorporation assay was performed to quantify cell proliferation. (*C*) WI-38 fibroblasts were transfected with the XRE-pNL1.3 [SecNLuc] plasmid. After 24 hours, cells were treated for 1 day with tryptophane (81 μM) in the presence or absence of epacadostat (15 μM). Untreated cells served as control. Cells were collected and AhR activity was measured by luciferase assay. Values were normalized based on total protein content. *(D)* WI-38 cells were treated with either 81 μM kynurenin (Kyn) or 81 μM kynurenic acid (KynA) for 10 days. Untreated cells served as control. Cells were subjected to senescence-associated β-galactosidase activity staining. Quantification is shown. *(E)* WI-38 fibroblasts were treated with kynurenin (Kyn; 81 μM) for 10 days. Untreated cells were used as control. Cell proliferation was quantified by BrdU incorporation assay. (*F*) WI-38 fibroblasts were transfected with the XRE-pNL1.3 [SecNLuc] plasmid. After 24 hours, cells were treated for 1 day with 81 μM KynA. Untreated cells served as control. Cells were collected and AhR activity was measured by luciferase assay. Values were normalized based on total protein content. Values represent means ± St Dev; statistical comparisons were made using 1-way ANOVA with post hoc test in A-D; statistical comparisons were made using the student's t-test in E and F. ***<0.001, ****<0.0001.

Figure S5. Downregulation of IL4I1 inhibits ATP-induced AhR activation and senescence. *(A-B)* WI-38 fibroblasts were transfected with either CTL or IL4I1 siRNA #2. After 24 hours, cells were treated with 1.5 mM ATP for 1 day. Untreated cells served as control. IL4I1 mRNA (*A*) and IL4I1 protein (*B*) expression was quantified by RT-PCR and immunoblotting analysis, respectively. Expression of GAPDH in (*A*) served as control. Ponceau S staining in (*B*) shows equal total protein loading. Quantification of protein band intensity is shown at the bottom of the blot. *(C)* WI-38 fibroblasts were transfected with either CTL or IL4I1 siRNA #2. After 24 hours, cells were transfected with the XRE-pNL1.3 [SecNLuc] plasmid. After 24 hours, cells were treated with 1.5 mM ATP for 1 day. Untreated cells served as control. Cells were collected and AhR activity was measured by luciferase assay. Values were normalized based on total protein content. *(D,E)* WI-38 fibroblasts were transfected with either CTL or AhR siRNA #2. After 24 hours, cells were treated with 1.5 mM ATP for 10 days. Untreated cells served as control. In (D), cells were subjected to senescence-associated β-galactosidase activity staining. In *(E)*, cells were collected and p21 protein expression was quantified by immunoblotting analysis using an antibody probe specific for p21. Ponceau S staining shows equal total protein loading. Quantification of protein band intensity is shown at the bottom of the blot. The Ponceau S staining is shared between panels *(B)* and *(E)*, as both panels originate from the same membrane probed sequentially with different antibodies. Values in C and D represent means ± St Dev; statistical comparisons were made using 2-way ANOVA with post hoc test. ****<0.0001.

Figure S6. I3P stimulation promotes senescence in an AhR-dependent manner. *(A,B)* WI-38 human diploid fibroblasts were treated with 27 μM I3P for 10 days. DMSO-treated cells served as control (vehicle). SA cell morphology was quantified in *(A)*, cell proliferation was quantified by BrdU incorporation assay in *(B)*. (*C*) WI-38 fibroblasts were treated for 1 day with 27 μM I3P. DMSO-treated cells served as control. AhR mRNA expression was quantified by RT-PCR. Expression of GAPDH served as control. *(D,F)* WI-38 cells were treated with either DMSO (vehicle) or 27 μM I3P for 10 days in the presence or absence of 10 μM BAY-218. SA cell

morphology was quantified in *(D)*, cell proliferation was quantified by BrdU incorporation assay in *(E)*, cell survival was quantified by MTT assay in (*F*). Values in A, B, and D-F represent means ± St Dev; statistical comparisons were made using 1-way ANOVA with post hoc test in D-F; statistical comparisons were made using the student's t-test in A and B. **<0.01, ****<0.0001.

Figure S7. ATP, tapinarof, and I3P stimulation promotes senescence in IMR-90 fibroblasts. *(A)* IMR-90 fibroblasts were treated with 1.5 mM ATP for 1 day. Untreated cells were used as control. Cells were collected and RNA extracted. IL4I1 mRNA level was determined by RT-PCR analysis. Expression of GAPDH served as control. *(B)* IMR-90 fibroblasts were treated with either 1.5 mM ATP, 27 μM I3P, or 9 μM tapinarof for 10 days. Untreated cells were used as control. Senescence was quantified by senescence-associated β-galactosidase activity. (*C*) WI-38 fibroblasts were treated with 27 μM I3P for 2 days, washed, and cultured for an additional 8 days. Senescence was quantified by senescence-associated β-galactosidase activity. (*D*) IMR-90 fibroblasts were treated with 27 μM I3P for 10 days to induce senescence. Untreated cells were used as control. Conditioned medium was used to culture MDA-MB-231 breast cancer cells for 48 hours. Cell proliferation was quantified by BrdU incorporation assay. (*E*) WI-38 fibroblasts were treated with 27 μM I3P for 2 days, washed, and cultured for an additional 8 days. Their conditioned medium was used to culture for 1 day MDA-MB-231 cells 24 hours after they were transfected with the XRE-pNL1.3 [SecNLuc] plasmid. Conditioned medium from untreated WI-38 cells served as control. MDA-MB-231 cells were collected and AhR activity was measured by luciferase assay. Values were normalized based on total protein content. (*F,G*) MDA-MB-231 cells were treated with either 1.5 mM ATP (*A*) or 27 μM I3P (*B*) for 10 days. Senescence was quantified by senescence-associated β-galactosidase activity. Values in B-G represent means ± St Dev; statistical comparisons were made using 1-way ANOVA with post hoc test in B; statistical comparisons were made using the student's t-test in C-G. ****<0.0001.
